# The negotiated equilibrium model of spinal cord function

**DOI:** 10.1113/JP275532

**Published:** 2018-07-10

**Authors:** Jonathan R. Wolpaw

**Affiliations:** ^1^ National Center for Adaptive Neurotechnologies, Wadsworth Center NYS Department of Health Albany NY USA; ^2^ Department of Neurology Stratton VA Medical Center Albany NY USA; ^3^ Department of Biomedical Sciences School of Public Health SUNY Albany NY USA; ^4^ Department of Neurology, Neurological Institute Columbia University New York NY USA

**Keywords:** spinal cord, motor learning, plasticity

## Abstract

The belief that the spinal cord is hardwired is no longer tenable. Like the rest of the CNS, the spinal cord changes during growth and ageing, when new motor behaviours are acquired, and in response to trauma and disease. This paper describes a new model of spinal cord function that reconciles its recently appreciated plasticity with its long‐recognized reliability as the final common pathway for behaviour. According to this model, the substrate of each motor behaviour comprises brain and spinal plasticity: the plasticity in the brain induces and maintains the plasticity in the spinal cord. Each time a behaviour occurs, the spinal cord provides the brain with performance information that guides changes in the substrate of the behaviour. All the behaviours in the repertoire undergo this process concurrently; each repeatedly induces plasticity to preserve its key features despite the plasticity induced by other behaviours. The aggregate process is a negotiation among the behaviours: they negotiate the properties of the spinal neurons and synapses that they all use. The ongoing negotiation maintains the spinal cord in an equilibrium – a negotiated equilibrium – that serves all the behaviours. This new model of spinal cord function is supported by laboratory and clinical data, makes predictions borne out by experiment, and underlies a new approach to restoring function to people with neuromuscular disorders. Further studies are needed to test its generality, to determine whether it may apply to other CNS areas such as the cerebral cortex, and to develop its therapeutic implications.

## Introduction

The spinal cord, together with its analogous brainstem nuclei, is the principal interface between the brain and the world. Spinal motoneurons control the muscles that produce behaviour, and spinal sensory afferents provide much of the information that guides it. Throughout life, as new behaviours are acquired and as growth and ageing occur, the spinal cord continues to perform its essential interface function with great reliability. Until recently, this reliability was explained by the assumption that the spinal cord is hardwired, that its neurons and synapses do not change once early development is completed. This traditional assumption is no longer viable.

Over the past several decades, it has become clear that the spinal cord, like the rest of the central nervous system (CNS), undergoes activity‐dependent change (i.e. plasticity) throughout life – with growth and ageing, as new behaviours are acquired, and in response to trauma and disease (Mendell, 1984; Wolpaw & Tennissen, 2001; Pierrot‐Deseilligny & Burke, 2012; Wolpaw, 2012; Christiansen *et al*. 2017). The recent recognition of this continual spinal cord plasticity raises a critical new question. When the spinal cord changes, how are existing behaviours maintained? Why do the ongoing changes in spinal neuronal and synaptic properties not disrupt the many behaviours for which the spinal cord is the final common pathway?

The behaviours most likely to be affected by spinal cord plasticity are effector‐specific. That is, they depend on specific contributions by specific populations of spinal motoneurons and their associated musculoskeletal apparatus. For example, human locomotion depends on, and is defined in terms of, well‐characterized rhythmic activity of the lumbosacral motoneurons that control leg movement and the cervical and thoracic motoneurons that control the associated arm and trunk movements (Ceccato *et al*. 2009; Zehr *et al*. 2009). Commonly referred to as ‘motor behaviours’, these behaviours are distinguished from ‘cognitive behaviours’, such as recounting the day's events or composing a poem, which might be accomplished by speaking, writing or signing, or even by coded foot‐stamping or eye‐blinking. Cognitive behaviours are not tied to or defined by specific contributions from specific motoneuron populations.

Because they do depend on the properties of specific spinal neurons and synapses, motor behaviours may be affected by the spinal cord plasticity associated with new motor behaviours, growth and ageing, or other life events. For example, the decreases in spinal reflexes associated with ballet training (Nielsen *et al*. 1993) or their changes with normal ageing (Koceja *et al*. 1995; Kido *et al*. 2004) may affect the participation of these reflex pathways in walking. Nevertheless, in normal life, previously acquired motor behaviours, like walking, are preserved despite subsequent spinal cord plasticity. While this preservation is certainly fortunate, it is also mysterious. Given a continually changing spinal cord, how are old motor behaviours preserved?

Two hundred years ago, the ancient idea that the spinal cord is simply a big nerve was replaced by the concept of a hard‐wired reflex centre that has prevailed up to the present (for historical review, Liddell, 1960; Neuburger, 1981; Clarke & Jacyna, 1987; Clarke & O'Malley, 1996). That concept is no longer adequate. This paper is the first formal explication and defence of a new model of spinal cord function that reconciles its newly appreciated plasticity with its long recognized reliability. (Brief summaries of the model have appeared previously, e.g. Wolpaw (2010) and Thompson and Wolpaw (2015).) The first section reviews the need for a new model – the now abundant evidence for life‐long spinal cord plasticity and the implications of this evidence. The second section defines the *negotiated equilibrium* model. The third section describes the evidence supporting it. The final section considers the model's practical and theoretical implications, the possibility that it applies elsewhere in the CNS, its relations to other CNS models, and the kinds of studies that are needed to test it further.

## Activity‐dependent plasticity in the spinal cord

### Spinal cord plasticity in pathological situations and reduced preparations

The long‐term changes in human spinal cord function that occur when an injury disturbs or eliminates supraspinal influence have been recognized for at least a century (Riddoch, 1917; Brodal, 1981; Hiersemenzel *et al*. 2000; Wolpaw & Tennissen, 2001). Deprived of the brain's influence and subjected to abnormal sensory inputs, spinal proprioceptive, nociceptive and autonomic pathways undergo changes that contribute to spasticity, contractures, renal disease, skin breakdown and autonomic dysregulation, which, in the absence of effective countermeasures, lead to extreme disability or death. Indeed, the long‐term survival and reasonable quality of life that are now possible after spinal cord injury are due in large part to meticulous bladder, bowel and skin‐care regimens that moderate and guide this spinal cord plasticity (Cardenas & Hooton, 1995; Henzel *et al*. 2011; Juknis *et al*. 2012).

**Figure 1:**
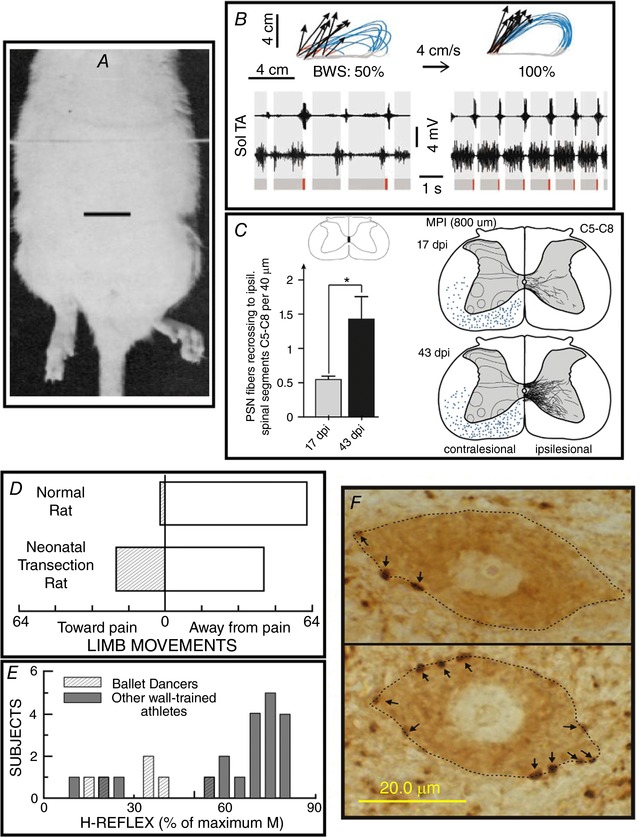
Six representative examples of activity‐dependent spinal cord plasticity. *A*–*C*, three examples illustrating spinal cord plasticity in pathological situations or in reduced preparations; *D*–*F*, three examples illustrating spinal cord plasticity in normal life. *A*, a hindlimb postural asymmetry produced by a unilateral cerebellar lesion persists after complete transection of the thoracic spinal cord. The cerebellar lesion occurred 60 min prior to the spinal cord transection. Scale bar, 2 cm. Modified from Chamberlain *et al*. (1963). *B*, impact of a combined treatment of serotonergic agonists, epidural electrical stimulation and locomotor training on treadmill locomotion (i.e. at 4 cm s^−1^) in spinal cord‐transected rats. Data from an untreated and a treated rat are shown on the left and right, respectively. Top: limb end‐point trajectories (with red indicating the initial drag phase of swing) and vectors representing the direction and magnitude of limb end‐point velocity at swing onset. The rat's percentage of body weight support (BWS) is indicated. Bottom: sequences of EMG activity from tibialis anterior (TA) and soleus (Sol) muscles. Grey and red bars indicate the stance and drag phases, respectively. Locomotion is far more normal, effective and consistent in the treated rat. Modified from Courtine *et al*. (2009), Nature Publishing Group, with permission. *C*, in the weeks following a C4 spinal hemisection in a rat, ipsilateral propriospinal neurons located above the lesion send axons into the contralateral spinal cord that descend to below the lesion and recross the midline at C5–8 to make functional connections. Left: the number of recrossing fibres increases greatly from 17 to 43 days post‐injury (dpi). Right: illustration of the marked increase in labelled recrossing fibres from 17 to 43 dpi. From Filli *et al*. (2014), Society for Neuroscience, with permission. *D*, the direction of flexion withdrawal responses to painful stimuli in normal adult rats and in adult rats in which the spinal cord was transected just after birth. In normal adults, the direction of the response is almost always correct (i.e. the limb moves away from the painful stimulus), while in transected adults it is often incorrect (i.e. the limb moves towards the stimulus). Neonatal spinal cord transection abolishes the descending input that gradually shapes normal (i.e. correct) flexion withdrawal responses. Modified from Levinsson *et al*. (1999), Society for Neuroscience, with permission. *E*, soleus H‐reflexes are much smaller in professional ballet dancers than in other well‐trained athletes (e.g. runners, swimmers and cyclists). (H‐reflexes of sedentary subjects fall in between.) The dancers’ smaller reflexes appear to be an important component of their skill acquisition. Modified from Nielsen *et al*. (1993), Springer Publishing, with permission. *F*, soleus motoneurons (dotted lines) from a control rat (top) and a rat in which the soleus H‐reflex was reduced by an operant down‐conditioning protocol (bottom). Arrows point to GABAergic terminals on the somatic membrane. The terminals are identified by glutamic acid decarboxylase (GAD67) immunoreactivity. After down‐conditioning, soleus motoneurons have many more GABAergic terminals, and these terminals are more densely labelled and cover more of the somatic membrane. The increase in GABAergic terminals is likely to be a component of the spinal cord plasticity that produces the smaller H‐reflex. Scale bar, 20 µm. From Wang *et al*. (2006), Wiley, with permission.

Ninety years ago, Anna DiGiorgio (1929, 1942) provided impressive laboratory evidence for activity‐dependent spinal cord plasticity by demonstrating that a brief period of abnormal descending activity produced a lasting change in spinal cord function. This striking phenomenon, labelled ‘spinal fixation’, was subsequently confirmed and elaborated by others (e.g. Chamberlain *et al*. 1963; Patterson, 1976). It is illustrated in Fig. 1 *A*.

Sixty‐five years ago, Shurrager and Dykman (1951) showed in cats that the isolated spinal cord could learn to walk better with training. In the 1980s, this phenomenon began to draw sustained interest (Lovely *et al*. 1986; Barbeau & Rossignol, 1987). Ongoing studies are identifying the underlying anatomical and physiological mechanisms, exploring methods for facilitating and enhancing this plasticity, and developing clinical applications (Courtine *et al*. 2009; Edgerton & Roy, 2009; Rossignol & Frigon, 2011; Rossignol *et al*. 2011; Harkema *et al*. 2012; e.g. Fig. 1 *B*). Other studies have documented and are exploring classical and operant conditioning in the isolated spinal cord (Horridge, 1962; Durkovic & Damianopoulos, 1986; Grau, 2013).

The energy and excitement that now characterize spinal cord injury research arise largely from growing understanding of the spinal cord's intrinsic capacities for plasticity. By enabling, augmenting and guiding these capacities, it should eventually be possible to replace lost neurons and preserve those that remain, to encourage appropriate axon regrowth, and to re‐establish effective synaptic connections (Fouad *et al*. 2011; Marsh *et al*. 2011; Becker & McDonald, 2012; Yoon & Tuszynski, 2012; Cregg *et al*. 2014; Liu *et al*. 2018; e.g. Fig. 1 *C*).

### Spinal cord plasticity in normal life

Spinal cord plasticity is not limited to pathological situations or reduced preparations; it is an essential part of normal life. Early in life, the acquisition of basic behaviours such as locomotion or withdrawal from a painful stimulus depends on spinal cord plasticity that is guided by the brain and by sensory input (Myklebust *et al*. 1982, 1986; Eyre *et al*. 2001; Martin *et al*. 2004; Schouenborg, 2008). Loss or distortion of these critical early influences (e.g. by perinatal damage to the brain) can lead to an abnormal adult spinal cord and sensorimotor deficits (Fig. 1 *D*). In these situations, methods for inducing more normal spinal cord plasticity early in life could improve adult function (e.g. Carmel *et al*. 2010).

Furthermore, spinal cord plasticity is important in the acquisition of motor behaviours throughout life. For example, professional ballet dancers have very weak stretch reflexes and H‐reflexes in their leg muscles (Fig. 1 *E*; Nielsen *et al*. 1993). This reflex depression probably contributes to the capacity to maintain the muscle coactivations essential in this form of dance, and thus it constitutes part of the CNS plasticity that underlies this complex athletic behaviour (Perez *et al*. 2007). Spinal proprioceptive reflexes change during the acquisition of more limited behaviours such as backward walking (Meyer‐Lohmann *et al*. 1986; Schneider & Capaday, 2003; Geertsen *et al*. 2008; Dragert & Zehr, 2011). They also change in the course of normal ageing (Koceja *et al*. 1995), which implies that the neural activity responsible for normal walking changes over the lifespan; walking is preserved but how it is produced changes. The success of rehabilitation training regimens in restoring function after spinal cord injury, stroke or in other disorders may depend significantly on the plasticity these regimens can induce in spinal reflex pathways (e.g. Thompson *et al*. 2013).

Operant conditioning protocols enable laboratory study and therapeutic application of spinal cord plasticity. In monkeys, rats, mice and humans, a protocol that bases reward on reflex size can produce changes in spinal reflex pathways similar to those occurring in normal life (Wolpaw *et al*. 1983; Wolpaw, 1987; Evatt *et al*. 1989; Chen & Wolpaw, 1995; Carp *et al*. 2006; Thompson *et al*. 2009). Over days and weeks, the protocol gradually creates multi‐site plasticity in brain and spinal cord that appears to function as a hierarchy: plasticity in the brain, acting through the corticospinal tract, induces plasticity in the spinal cord that directly underlies the larger (or smaller) reflex. The spinal cord plasticity includes changes in motoneuron intrinsic properties (e.g. firing threshold and axonal conduction velocity) and synaptic inputs, and in spinal interneurons (e.g. Fig. 1 *F*; reviewed in Wolpaw 2010; Thompson & Wolpaw, 2014). In addition to changing the reflex, this plasticity affects other behaviours, such as locomotion, that use these spinal neurons and synapses (Chen *et al*. 2005). Thus, appropriate reflex conditioning can improve locomotion after incomplete spinal cord injuries in animals and humans (Chen *et al*. 2006; Manella *et al*. 2013; Thompson *et al*. 2013). Conditioning protocols that target specific spinal pathways (i.e. protocols selected on the basis of the individual's specific functional deficits) are a promising new therapeutic approach to restoring function after spinal cord injury or other trauma or disease (Thompson & Wolpaw, 2015).

## The negotiated equilibrium model of spinal cord function

The *negotiated equilibrium* model is a response to the question of how the continually changing spinal cord remains a reliable common pathway throughout life. This section presents the three components of the model; the next section reviews the laboratory and clinical data that led to and support each of these components. The model is as follows.

### (a) The distributed substrate of a motor behaviour

Throughout life, the central nervous system acquires and maintains a repertoire of motor behaviours. Each new motor behaviour is acquired through a learning experience that induces plasticity in the brain that leads to plasticity in the spinal cord. Thus, the substrate of each behaviour is a hierarchy of plasticity: plasticity in the brain induces and maintains plasticity in the spinal cord. The brain and spinal cord plasticity combine to produce and preserve satisfactory performance. For each behaviour, satisfactory performance is defined by a set of key features. For example, the key features of locomotion include right‐left symmetry in step timing and in hip height. Each time the behaviour occurs, deviations from its key features (i.e. error signals) induce changes in its substrate that tend to reduce the deviations. In this process, the spinal cord provides the brain with performance information that guides appropriate changes in the hierarchy of brain and spinal cord plasticity underlying the behaviour. In sum, this first component of the negotiated equilibrium model proposes that the substrate of a motor behaviour actively maintains itself in the CNS.

### (b) Negotiation among the behaviours

All the motor behaviours in the repertoire undergo this maintenance process concurrently. Each behaviour operates as an independent agent that repeatedly induces spinal cord (and brain) plasticity to preserve its key features despite the plasticity induced by other behaviours. Thus, the aggregate process is a negotiation among the behaviours. They negotiate the properties of the spinal neurons and synapses that they all use. This ongoing negotiation maintains spinal neuronal and synaptic properties in an equilibrium – a negotiated equilibrium – that serves all the behaviours in the repertoire. When the acquisition of a new behaviour changes the spinal cord, it begins a new negotiation that includes the new behaviour and all the old behaviours. The outcome is a new spinal cord equilibrium that serves all the behaviours in the expanded repertoire. While this expanded negotiation preserves the key features of old behaviours, it may change their muscular and kinematic details. For example, the muscle activations and joint rotations that produce satisfactory locomotion may change. A new negotiation may also occur when the spinal cord is modified by growth or ageing, when peripheral changes (e.g. in limb length or body weight) alter sensory inputs or the kinematic impact of motor outputs, or when trauma or disease impairs old behaviours. In the case of CNS trauma or disease, the negotiation often fails to fully restore the key features of old behaviours, and some impairment remains (e.g. limping after an incomplete spinal cord injury). In all these situations, the occurrence of an effective negotiation requires that the brain receive information on the performance of each behaviour.

### (c) Potential therapeutic value of a new motor behaviour

When an old behaviour is impaired, new learning that targets appropriate plasticity to a spinal site important in the old behaviour can improve that behaviour. Furthermore, by modifying an important site and by joining the ongoing negotiation among the behaviours, the new behaviour can lead to plasticity at other sites that further improves the old behaviour. That is, by moving the current state of the spinal cord away from its previous location in the multidimensional space comprising the values of all spinal neuronal and synaptic properties, the new behaviour can enable the old behaviour to escape an inferior solution (i.e. a local minimum) reached prior to the new learning, and to thereby more nearly restore its key features. Thus, new learning that induces targeted plasticity could supplement less specific therapies and enhance recovery of motor function.

## Evidence supporting the model

Much of the data leading to the negotiated equilibrium model have been obtained with a simple example of motor learning that takes advantage of the simplicity of the spinal cord and its distance from the rest of the CNS. The spinal stretch reflex (SSR) (e.g. the ‘knee‐jerk’ reflex), and its electrical analogue, the H‐reflex, are mediated by a wholly spinal pathway that conveys proprioceptive input to spinal motoneurons (Magladery *et al*. 1951; Matthews, 1972; Baldissera *et al*. 1981; Henneman & Mendell, 1981; Brown, 1984; Pierrot‐Deseilligny & Burke, 2012). This pathway comprises group Ia afferent fibres, their monosynaptic contacts on spinal motoneurons and the motoneurons. In addition, it may include groups II and Ib afferent fibres; and the afferents may also reach the spinal motoneurons via one or two intervening spinal interneurons.

**Figure 2:**
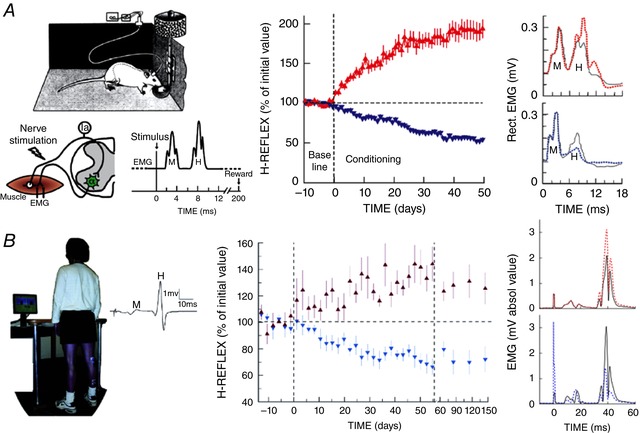
A simple motor behaviour: the reflex operant conditioning protocol and its results in rats (*A*) and humans (*B*) *A*, left: soleus EMG is monitored 24 h per day in a rat with implanted EMG electrodes and a tibial nerve cuff. The wires travel subcutaneously to a head‐mounted connector and through a flexible cable and a commutator to amplifier and stimulator. The rat can move freely about the cage. The diagram shows the main pathway of the H‐reflex (i.e. the Ia afferent fibre and the spinal motoneuron). (Other large afferents and di‐ or trisynaptic paths may also contribute to the H‐reflex.) Whenever the absolute (i.e. rectified) value of soleus EMG activity stays in a specific range for a randomly varying 2.3–2.7 s period, a nerve cuff stimulus elicits a threshold M wave (a direct muscle response) and an H‐reflex. The trace illustrates one trial. A rat averages 2000–6000 trials day^−1^. Middle: for Days −10 to 0, the rat is exposed to the control mode, in which no reward occurs and the H‐reflex is simply measured to define its initial size. For Days 0–50, it is exposed to the HR_up_ or HR_down_ mode, in which a food‐pellet reward occurs when the H‐reflex is above (HR_up_) or below (HR_down_) a criterion. Background EMG activity and M wave are stable throughout. Successful conditioning (change of at least 20% in the correct direction) occurs in more than 80% of the rats (the other rats remain within 20% of their initial value). The graphs show average (±SEM) daily H‐reflex sizes for 59 successful HR_up_ rats (red) and 81 successful HR_down_ rats (blue). In both, mode‐appropriate H‐reflex change develops steadily over the 50 days. If the mode switches from HR_up_ to HR_down_ mode (or vice versa) (not shown), the change reverses in the same gradual fashion. Right: average post‐stimulus EMG activity (absolute value) for a day from an HR_up_ rat (top) and an HR_down_ rat (bottom) in control mode (continuous line) and after conditioning (dashed line). The H‐reflex is larger after up‐conditioning and smaller after down‐conditioning. Background EMG activity (shown here by EMG at time zero) and M waves are not changed. From Wolpaw (2010) and Wolpaw (1997), Elsevier, with permission. *B*, left: soleus EMG is monitored in a person with EMG electrodes over the muscle. The person stands facing a screen showing the current level (absolute value) of soleus EMG activity *versus* a specified range. When EMG stays in the range for several seconds, stimulation of the tibial nerve by electrodes in the popliteal fossa elicits a threshold M wave and an H‐reflex. The trace is one trial (showing unrectified EMG activity). Middle: a person completes three 225‐trial sessions per week. The first 6 sessions (Days −14 to 0) are in the control mode, in which the H‐reflex is simply measured to define its initial size. The next 24 sessions (Days 0–56) are in the HR_up_ or HR_down_ mode, in which the screen gives immediate feedback after each trial as to whether the H‐reflex was above (HR_up_) or below (HR_down_) a criterion. After these 24 sessions, the person returns for four follow‐up sessions over 3 months. Background EMG activity and M wave are stable throughout. Conditioning is successful in ∼80% of the people. The graphs show average (±SEM) daily H‐reflex sizes for 6 successful HR_up_ (red) and 8 successful HR_down_ (blue) people. In both, H‐reflex change develops steadily over the 24 conditioning sessions. In follow‐up sessions, the H‐reflex increase in the HR_up_ group is smaller but still evident, and the decrease in the HR_down_ group is unchanged. Right: average post‐stimulus EMG activity for a session from an HR_up_ person (top) and an HR_down_ person (bottom) in control mode (continuous line) and at the end of conditioning (dotted line). The H‐reflex is larger after up‐conditioning and smaller after down‐conditioning. Background EMG and M waves do not change. (Stimulus artifacts at 0 ms.) From Thompson & Wolpaw (2014) and Thompson *et al*. (2009).

This spinal pathway is influenced by descending activity from the brain. As noted above in the review of spinal cord plasticity, an operant conditioning protocol that modifies this descending activity can increase or decrease the SSR or H‐reflex (Wolpaw *et al*. 1983; Wolpaw, 1987; Evatt *et al*. 1989; Chen & Wolpaw, 1995; Carp *et al*. 2006; Thompson *et al*. 2009); for review, Thompson & Wolpaw 2014). This phenomenon has been demonstrated in monkeys, rats, mice and humans. The reflex changes gradually over days and weeks, and the change reverses in the same gradual fashion when the reward criterion is reversed. Figure 2 illustrates operant conditioning of the soleus H‐reflex in rats and in humans, and summarizes its results. The larger or smaller SSR or H‐reflex produced by this protocol is a simple motor skill, *an adaptive behaviour acquired through practice* (Compact Oxford English Dictionary, 1993; Shmuelof & Krakauer, 2011). In everyday life, similar adaptive changes in reflex pathways are components of complex motor skills (e.g. Nielsen *et al*. 1993; Schneider & Capaday, 2003).

A long series of studies have explored the spinal cord and brain plasticity associated with the acquisition of this simple skill, and have examined the impact of that acquisition on a previously acquired motor behaviour – locomotion. Together with a variety of other laboratory and clinical studies, the results support the three components of the negotiated equilibrium model: (a) the distributed substrate of a motor behaviour; (b) negotiation among the behaviours; and (c) the potential therapeutic value of a new behaviour. The evidence supporting each of these three components is described below.

### The distributed substrate of a motor behaviour

The separation of the spinal cord from the brain and their connection through experimentally accessible tracts have made it possible to show that motor learning depends on plasticity in both structures and to explore how brain and spinal cord plasticity interact to produce a new behaviour.

**Figure 3:**
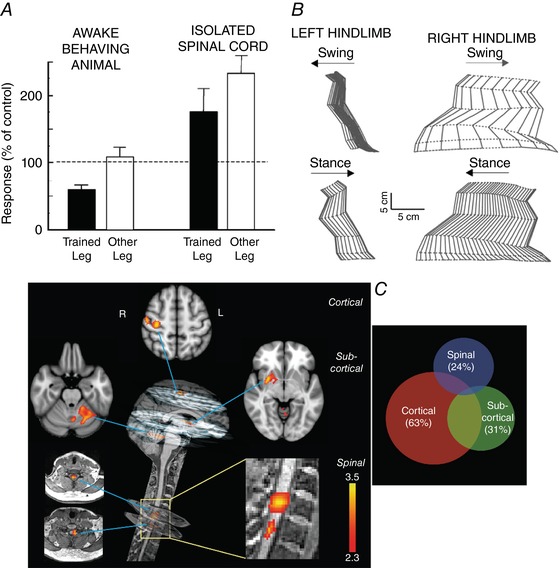
The substrates of motor behaviours are distributed between brain and spinal cord *A*, reflex responses from monkeys after H‐reflex down‐conditioning. Left side: average triceps surae H‐reflexes of awake behaving monkeys in the down‐conditioned (i.e. trained) leg and the other leg. The H‐reflex in the trained leg is much smaller than its control value, while the H‐reflex in the other leg is not changed from control. Right side: average maximum triceps surae monosynaptic reflex responses to dorsal root stimulation from the same monkeys under anaesthesia and after mid‐thoracic spinal cord transaction. The reflex asymmetry created by down‐conditioning is still present, but the reflexes in both legs are larger than those from the isolated spinal cords of control (i.e. unconditioned) monkeys. The contrast between the results from the awake behaving animals and the results from their isolated spinal cords indicates that the H‐reflex down‐conditioning protocol produced plasticity in the brain and on both sides of the spinal cord. Data from Wolpaw and Lee (1987, 1989) and Wolpaw *et al*. (1993). *B*, joint angles of the left and right hindlimbs during the swing and stance phases of clonidine‐induced locomotion 72 days after spinalization in a cat in which the left lateral gastrocnemius and soleus (LGS) muscles had been denervated 49 days before spinalization. During the 49 days prior to spinalization, locomotion had recovered from the impairment produced by the denervation. To prevent overlap, individual stick figures are displaced by an amount equal to the displacement of the foot along the horizontal axis. Left and right hindlimbs are viewed from the left and right sides, respectively. Horizontal arrows indicate the direction of hindlimb movement during the stance and swing phases. Spinal locomotion is greatly impaired on the left side (i.e. the denervated side), indicating that the spinal cord had changed during the pre‐spinalization compensation following the left LGS denervation. From Frigon and Rossignol (2009), Elsevier, with permission. *C*, neural correlates of motor sequence learning (i.e. a specific sequence of individual finger flexions). Left: distinct cortical, subcortical and spinal clusters show learning‐related modulation in activity during the motor sequence learning condition. All activation clusters correlate positively with performance speed. The cortical activation cluster is in the contralateral sensorimotor cortex. At the subcortical level, one cluster is in the contralateral putamen, while the other is in lobule V–VI of the ipsilateral cerebellum. The spinal cord activation clusters are centred in the C7 and C8 spinal segments. (The colour bars indicate *Z*‐score values; all activation maps are corrected for multiple comparisons.) Right: activity in both the spinal cord and the brain accounts for non‐overlapping portions of the variability in performance speed. The Venn diagram illustrates the amount of performance variability that is explained by cortical, subcortical or spinal cord activity independently, as well as their shared variance. The numbers in parentheses are the percentage of total variance explained by the activity in each region. Brain and spinal cord plasticity make independent contributions to performance. From Vahdat *et al*. (2015).

When the monkey triceps surae H‐reflex is down‐conditioned in one leg, the contralateral triceps surae H‐reflex does not change (Wolpaw *et al*. 1993). However, when the animal is anaesthetized and complete transection of the thoracic spinal cord removes the brain's influence, the surprising picture summarized in Fig. 3 *A* emerges. Although the reflex asymmetry created by the conditioning protocol is still evident, the reflex on the down‐conditioned side is larger than that of a naïve animal, and the contralateral reflex is even larger (Wolpaw & Lee, 1989). Together with the extensive evidence for plasticity on the down‐conditioned side of the spinal cord (Wolpaw, 2010; Thompson & Wolpaw 2014), this unexpected result indicates that unilateral down‐conditioning changes both sides of the spinal cord, and the brain as well. In the awake animal, brain and spinal cord plasticity combine to produce a smaller reflex on the conditioned side and a normal reflex on the contralateral side. When the brain's influence is removed, the spinal cord plasticity alone affects function, and the picture shown in Fig. 3 *A* appears.

Experiments in cats by Rossignol and colleagues provide further examples of how brain and spinal cord plasticity may combine to produce a motor behaviour. Carrier *et al*. (1997) compared the locomotor impact of spinal cord transection followed by unilateral denervation of ankle flexor muscles to the impact of the denervation followed by the transection. When the sequence was (1) spinal transection, (2) treadmill training, (3) denervation and (4) more treadmill training, locomotion returned to nearly normal. Increases in hip and knee flexion compensated for the decreased ankle flexion caused by the denervation. In contrast, when the sequence was (1) denervation, (2) treadmill training, (3) spinal transection and (4) more treadmill training, locomotion never recovered. The increases in hip and knee flexion that had followed denervation alone increased further and other marked abnormalities in muscle activity appeared. Frigon and Rossignol (2009) extended this work and delineated the effect of pre‐transection denervation on the post‐transection evolution of spinal reflexes. The deleterious impact of prior denervation on locomotor recovery after subsequent spinal transection indicates that the denervation had induced changes in both the spinal cord and the brain that together accounted for the initial return of nearly normal locomotion. After spinal cord transection removed the influence of the brain, the spinal cord plasticity functioned in isolation, and the result was grossly abnormal locomotion. Figure 3 *B* illustrates this striking effect. As with down‐conditioning of the H‐reflex (Fig. 3 *A*), the locomotor recovery after denervation and before spinal cord transection was due to the combined impact of plasticity in both brain and spinal cord.

In a recent functional magnetic resonance (fMRI) study, Vahdat *et al*. (2015) examined brain and spinal cord activity during motor sequence learning. They found that the learning correlated with changes in cortical, cerebellar and spinal activity. As learning progressed, the spinal activity became less correlated with the cortical activity and negatively correlated with the cerebellar activity. Most significant in the present context, activity in each of the three CNS regions made an independent contribution to skill acquisition. This result indicates that motor sequence learning, which is traditionally ascribed to plasticity in the brain, also depends on plasticity in the spinal cord. Figure 3 *C* summarizes this important finding.

A series of lesion studies have taken advantage of the separation between the brain and the spinal cord to explore how changes in both places combine to create and maintain a smaller H‐reflex. There are three major findings. The first is that the corticospinal tract (CST) is essential for H‐reflex down‐conditioning; other major descending tracts are not needed (Chen & Wolpaw, 1997, 2002). If the CST is transected prior to down‐conditioning, the H‐reflex does not decrease; if it is cut after down‐conditioning has occurred, the H‐reflex decrease disappears over 5–10 days. Thus, down‐conditioning depends on spinal cord plasticity that is guided by the brain through the CST and can survive 5–10 days without continued CST influence. Several lines of evidence suggest that this influence is not limited to the periods of the conditioning trials; rather, it is likely to be continuous. In laboratory animals, the magnitude of H‐reflex change is not correlated with the number of trials per day (Wolpaw *et al*. 1993). Furthermore, people who perform only 3–5% as many trials as animals change the H‐reflex nearly as much (Thompson *et al*. 2009). Furthermore, while a conditioned H‐reflex decrease disappears in 5–10 days after CST transection, a conditioned decrease or increase persists for weeks or longer after simple cessation of the conditioning protocol (Wolpaw *et al*. 1986; Thompson *et al*. 2013). This suggests that the critical CST influence continues for some time after conditioning ceases.

The second major finding is that cerebellar output to cortex is also essential for down‐conditioning. If the main cerebellar output nuclei, the dentate and interpositus nuclei (DIN), are ablated prior to down‐conditioning, the H‐reflex does not decrease. If they are ablated after down‐conditioning has occurred, some of the decrease disappears immediately while the remainder survives for 40 days and then disappears (Chen & Wolpaw, 2005; Wolpaw & Chen, 2006). These results, together with the fact that transection of the rubrospinal tract (i.e. the principal cerebellar output to the spinal cord) does not impair down‐conditioning (Chen & Wolpaw, 1997, 2002), imply that the cerebellum's output to cortex is its essential contribution. The long (40‐day) delay after DIN ablation before loss of the H‐reflex decrease, compared to the short (5–10 day) delay after CST transection, implies that the essential CST activity depends on plasticity in sensorimotor cortex (the origin of the CST) or in closely related areas, that can survive for 40 days without continued cerebellar influence.

The third major finding concerning how brain and spinal plasticity combine to change the H‐reflex is that inferior olive (IO) output to cerebellum is essential for down‐conditioning. If the IO is ablated before down‐conditioning, the H‐reflex does not decrease; if it is ablated after down‐conditioning, some of the decrease disappears over 10 days while the remainder survives for 40 days and then disappears (Chen *et al*. 2016*a*,*b*). The gradual partial loss of the H‐reflex decrease after IO ablation compared to the immediate partial loss after DIN ablation suggests that the IO guides cerebellar plasticity that survives 10 days without continued IO influence.

**Figure 4:**
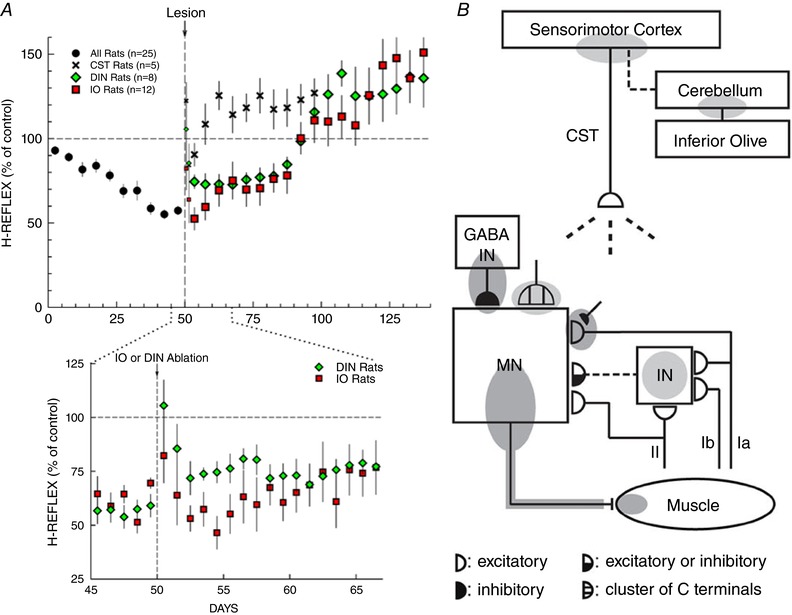
A hierarchy of brain and spinal cord plasticity underlies a simple motor behaviour *A*, effects of different lesions (corticospinal tract (CST) transection, dentate and interpositus nuclei (DIN) ablation, or inferior olive (IO) ablation) on maintenance of H‐reflex down‐conditioning. CST data from Chen & Wolpaw (2002); DIN data from Wolpaw and Chen (2006); IO data from Chen *et al*. (2016*b*). Top: average H‐reflex sizes (±SEM) for each 5‐day period for the first 50 days of down‐conditioning for all 25 rats and for the next 50–100 days for CST‐transected rats (*n* = 5), DIN‐ablated rats (*n* = 8) and IO‐ablated rats (*n* = 12). For the 5 days immediately post‐lesion, H‐reflex sizes are shown for the first day and the second day (smaller symbols), and for the next three days together. All the lesions show a transient non‐specific increase in the first 1–2 days due to the anaesthesia and/or the lesion procedure. After this brief non‐specific effect dissipates, all three lesions result eventually in an H‐reflex larger than its initial control size. In the CST rats, this final size is reached within 10 days; in the DIN and IO rats it is not reached until ∼50 days after the lesion. The H‐reflex down‐conditioning mode remains in effect throughout. Bottom: expansion showing average daily H‐reflex values for the IO and DIN rats for the days immediately before and after IO or DIN ablation. In the DIN rats, the H‐reflex increases ∼20% within the first 1–2 days after DIN ablation; in the IO rats, a similar increase develops over the first 10 days after IO ablation. (The brief non‐specific increase in the first 1–2 post‐ablation days is evident for both ablations.) *B*, the shaded ovals indicate the spinal and supraspinal sites of definite (dark grey) or probable (light grey) plasticity associated with operant conditioning of the H‐reflex. CST, the main corticospinal tract; GABA IN, a GABAergic spinal interneuron; IN, a spinal interneuron; MN, the motoneuron. Dashed pathways imply the possibility of intervening spinal interneurons. The monosynaptic and probably oligosynaptic H‐reflex pathway from groups Ia, II and Ib afferents to the motoneuron is shown. Definite or probable sites of plasticity include the following: the motoneuron membrane (i.e. firing threshold and axonal conduction velocity); motor unit properties; GABAergic interneurons; GABAergic terminals and C terminals on the motoneuron; the Ia afferent synaptic connection; terminals conveying oligosynaptic groups I and II inhibition or excitation to the motoneuron; sensorimotor cortex; and cerebellum. As described in the text, the data summarized in *A* and *B* suggest that the reward contingency acts through the inferior olive to guide and maintain plasticity in the cerebellum that guides and maintains plasticity in sensorimotor cortex that (via the CST) guides and maintains plasticity in the spinal cord that is directly responsible for H‐reflex change. Updated from Wolpaw and Tennissen (2001) and Wolpaw (2010).

The effects of DIN and IO ablation on H‐reflex down‐conditioning are consistent with current ideas about the role of the cerebellum and IO in other simple motor skills (Martin *et al*. 1996; Boyden *et al*. 2004; Thompson, 2005; Freeman & Steinmetz, 2011; Cheron *et al*. 2013; Longley & Yeo, 2014). Vestibuloocular reflex conditioning and eyeblink conditioning are thought to involve cerebellar plasticity caused by the conjunction of activity in specific mossy and climbing fibres, with the climbing fibres, which originate in the IO, providing a teaching signal (Boyden *et al*. 2004; Thompson, 2005; Welsh *et al*. 2005; Freeman & Steinmetz, 2011; Schonewille *et al*. 2011; Cheron *et al*. 2013; Longley & Yeo, 2014; Mauk *et al*. 2014). A similar conjunction could underlie H‐reflex conditioning. The mossy fibres could convey efference‐copy activity that reflects current CST influence over the H‐reflex pathway (Leergaard *et al*. 2006; Suzuki *et al*. 2012; Ruigrok *et al*. 2015), and climbing fibre activity could indicate whether a reward occurs (e.g. whether the IO receives input caused by the click of the pellet dispenser or the consumption of the food pellet) (for review of IO inputs, Ruigrok *et al*. 2015). Cerebellar plasticity resulting from this conjunction could produce output to sensorimotor cortex that increases the probability of CST activity that decreases the H‐reflex, and thereby increases the probability of reward. By providing evidence for cerebellar plasticity, the difference between DIN ablation and IO ablation in the rate of the initial post‐ablation H‐reflex increase (Fig. 4 *A*) supports this possibility.

Figure 4 *A* summarizes the effects of CST transection, DIN ablation and IO ablation on maintenance of a down‐conditioned H‐reflex. None of CST transection, DIN ablation or IO ablation has a long‐term effect on H‐reflex size in naïve (i.e. unconditioned) rats (Chen *et al*. 2001, 2016a; Chen & Wolpaw, 2005). Thus, the effects in Fig. 4 *A* are effects on the maintenance of a down‐conditioned H‐reflex. The three lesions arrive at the same final result over three different time courses. The differences suggest that the substrate of H‐reflex down‐conditioning is a hierarchy of plasticity: the IO maintains plasticity in the cerebellum that maintains plasticity in sensorimotor cortex (or related areas) that maintains CST activity that maintains plasticity in the spinal cord that is responsible for the smaller H‐reflex. The fact that all three lesions lead eventually to an H‐reflex larger than its initial size indicates that this hierarchy does not fully account for the impact of down‐conditioning. The origin and import of the large post‐lesion H‐reflex are considered elsewhere (Wolpaw & Lee, 1989; Wolpaw & Tennissen, 2001; Wolpaw, 2010; Chen *et al*. 2011).

Figure 4 *B* summarizes current knowledge of the brain and spinal cord plasticity underlying and associated with H‐reflex conditioning. Several aspects of this plasticity deserve emphasis. First, it includes changes in the spinal motoneuron, in several different synaptic populations on the motoneuron, and in spinal interneurons as well. Thus, H‐reflex conditioning is likely to affect other behaviours that use these spinal neurons and synapses. Second, H‐reflex down‐conditioning and up‐conditioning are not simply mirror images of each other; they involve different mechanisms to at least some degree (for review Wolpaw 2010; Thompson & Wolpaw, 2014). As to their dependence on connections to the brain, the acquisition of up‐conditioning, like that of down‐conditioning, requires the CST (Chen *et al*. 2002). While the post‐lesion H‐reflex increase (see above) complicates assessment of the CST dependence of up‐conditioning maintenance, the existing data suggest that up‐conditioning maintenance, unlike down‐conditioning maintenance, does not require the CST (Chen *et al*. 2003). Recent human studies have provided additional insight. They indicate that operantly conditioned H‐reflex change consists of task‐dependent adaptation that apparently reflects plasticity in the brain, plus long‐term change that reflects spinal cord plasticity (Thompson *et al*. 2009, 2013).

The evidence that the neural substrates of H‐reflex conditioning, functional recovery after peripheral denervation and motor sequence learning all have both cranial and spinal components suggests that the substrates of other motor behaviours are similarly distributed. While the cranial components of different behaviours might possibly involve different neurons and synapses (or even different brain areas), their spinal components – motoneuron and interneuron intrinsic properties and synaptic inputs – necessarily overlap. This inevitable overlap is the principal impetus and rationale for the negotiated equilibrium model.

This model postulates that the distributed substrate of plasticity underlying a motor behaviour changes as needed to ensure that the behaviour is maintained over time. What exactly is it maintaining? Most motor behaviours are kinematically complex, involving concurrent changes in multiple joint angles and in the positions of multiple limb segments. For each behaviour, some of these kinematic variables are more important than others. For example, in the rapid withdrawal of a finger tip from a hot stove, the exact changes in each of the many hand/arm joint angles can vary widely as long as their net effect is the rapid removal of the finger tip from the stove; the change in the vertical position of the finger tip is the important variable (i.e. the key feature). Bernstein (1967) emphasized that motor behaviours have key features that are much more precisely controlled than other variables. More recently, this principle has been formalized in the ‘uncontrolled manifold’ concept, which partitions the variance in kinematic variables into the part that impairs key features (e.g. finger‐tip vertical position relative to a hot stove) and the part that does not (Scholz & Schöner, 1999; Latash *et al*. 2007; Chang *et al*. 2009). In terms of this analysis, the maintenance of a behaviour means the preservation of its key features; this is the goal of the distributed substrate of plasticity underlying the behaviour. Evidence presented in the next subsection supports this idea.

### Negotiation among the behaviours

The negotiated equilibrium model uses the idea that certain variables are precisely controlled to explain the impact of a new motor behaviour on an old behaviour that employs the same spinal circuitry. It predicts that, when new learning changes the lumbosacral spinal cord, the variables that define normal locomotion (i.e. its key features) will be preserved. For example, these are likely to include right/left symmetry in step length and in hip height. Without these symmetries, the animal or human limps, the hips are tilted, the spine is twisted or bent laterally, and in the long term musculoskeletal damage may develop. Key features are also likely to include step length and metabolic cost. In contrast, other features, such as ankle, knee, and hip joint rotations and muscle activations over the step cycle, might change, and might even differ for right and left sides, as long as their collective result is the preservation of key features such as the right/left symmetries. In sum, the variables that account for a key feature may change, but the key feature does not.

The data support these predictions. When operant conditioning increases or decreases the rat soleus H‐reflex, right/left step symmetry and hip‐height symmetry are preserved, while muscle activation and joint angles change (Chen *et al*. 2005, 2011). In accord with the change in the H‐reflex, soleus EMG activity and locomotor ankle angle increase with up‐conditioning and decrease with down‐conditioning. At the same time, hip angle may decrease with up‐conditioning or increase with down‐conditioning; as a result, the hip height on the conditioned side does not become higher or lower, respectively, than that on the contralateral side (Chen *et al*. 2011). It appears that additional ‘compensatory’ plasticity prevents the change in ankle angle from impairing locomotion. Evidence for such additional plasticity includes the finding that up‐ or down‐conditioning of the soleus H‐reflex is usually accompanied by an opposite change (i.e. decrease or increase, respectively) in the vastus lateralis H‐reflex (Chen *et al*. 2011).

It seems probable that the set of spinal cord properties that change to support a new behaviour is defined in part by their impact on key features of old behaviours (Ajemian *et al*. 2013). Changes in properties that do not affect key features of old behaviours may be more likely to survive the negotiation between new and old behaviours than changes in properties that do affect them. However, in the highly constrained environment of the spinal cord, in which many behaviours share the same neurons and synapses, the capacity for such separation among the sets of properties supporting different behaviours is likely to be limited. Thus, the negotiation is likely to entail further plasticity that preserves the key features of old behaviours by changing how old behaviours are produced (i.e. by changing their EMG and kinematic details).

In the present context, the primary import of this further plasticity is that it compensates for the impact on old behaviours of the plasticity underlying the new behaviour; it thereby ensures that the key features of old behaviours are preserved. At the same time, it can also affect old behaviours in other ways. An excellent example is the distinctive walk of professional ballet dancers (Kilgannon, 1996). The changes in spinal reflex pathways (Nielsen *et al*. 1993) (and presumably in other pathways as well) that underlie the acquisition of this very specialized behaviour lead to modifications in the old behaviour of locomotion; dance training affects how ballet dancers walk. The substrate of brain and spinal plasticity underlying locomotion changes; it achieves the same key features through a different pattern of neuronal activity. Thus, a new behaviour may change the ongoing negotiation of spinal properties in two ways: first, by adding itself to the negotiation; and second, by changing some of the old behaviours that participate in the negotiation. Both kinds of changes will affect the impact of subsequent learning and are relevant to the therapeutic implications of the negotiated equilibrium model (see below).

According to the negotiated equilibrium model, the compensatory plasticity that prevents new learning from disrupting old behaviours depends on interactions between the spinal cord and the brain. Each time an old behaviour occurs, deviations from its key features induce changes in its substrate that tend to reduce the deviations. In this process, the spinal cord provides the brain with performance information that guides appropriate changes in the substrate of brain and spinal plasticity responsible for the old behaviour. (This guidance might occur via a mechanism in which properties repeatedly undergo random variations and only those that improve performance are retained (Reinkensmeyer *et al*. 2012).) If this picture is correct, transection of the ascending sensory pathways to the brain should prevent the compensatory plasticity that preserves old behaviours. New motor learning should then impair key features of old behaviours.

**Figure 5:**
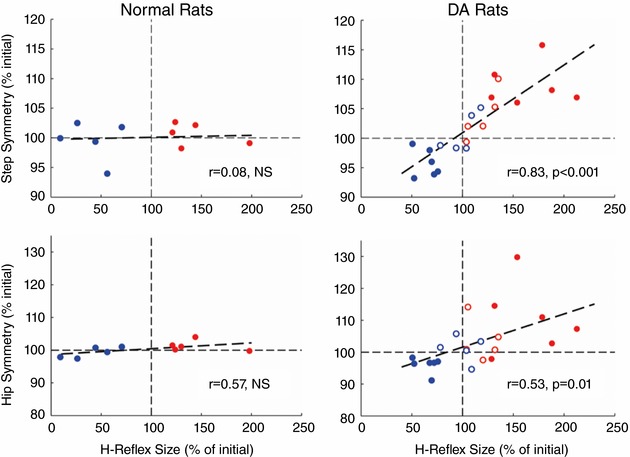
When a new motor behaviour changes the spinal cord, sensory feedback from spinal cord to brain guides the preservation of an old behaviour The graphs show the effects of soleus H‐reflex up‐conditioning (red) or down‐conditioning (blue) on right/left step symmetry and hip height symmetry in normal rats and in rats in which the dorsal ascending tracts have been transected at T8–9 (DA rats). Filled symbols represent final H‐reflex size for rats transected before H‐reflex recording; they show the percentage of H‐reflex size for the control mode period prior to conditioning. Open symbols represent final H‐reflex size for rats transected after 50 days of conditioning and before another 50 days of conditioning; they show the percentage of H‐reflex size at the end of the initial 50 days of conditioning (i.e. just before transection). The *y*‐axis shows final right–left symmetry. In normal rats, H‐reflex up‐ or down‐conditioning does not affect symmetry. In contrast, in DA rats, H‐reflex up‐ or down‐conditioning produces asymmetries that correspond to the direction and magnitude of H‐reflex change: the ratios of right‐step duration to left‐step duration and right hip height to left hip height increase with H‐reflex increase and decrease with H‐reflex decrease. As a result, the DA rats limp and tilt as they walk. From Chen *et al*. (2017).

This prediction was recently tested in rats in which mid‐thoracic transection of the spinal dorsal ascending tracts (i.e. DA transection) largely abolished proprioceptive feedback to the brain from the lumbosacral spinal cord (Chen *et al*. 2017). The lesion alone did not impair locomotor right–left symmetry in step timing or hip height. However, in these rats, up‐ or down‐conditioning of the right soleus H‐reflex produced corresponding asymmetries in step timing and hip height. Up‐conditioning prolonged the right stance period and elevated right hip height, while down‐conditioning did the opposite. Figure 5 illustrates the deleterious impact on step symmetry. The rats limped and had a tilted posture. As predicted by the model, the lack of sensory feedback prevented the compensatory changes that would normally have preserved normal locomotion despite the change in the H‐reflex pathway. The result was that the substrate of plasticity responsible for locomotion did not change to accommodate the new learning.

### The potential therapeutic value of a new motor behaviour

Previous sections addressed the impact of a new behaviour on an old behaviour that is fully intact prior to the new learning. For normal animals, the model predicts that compensatory plasticity will preserve the key features of the old behaviour. This is indeed what occurs. However, if the old behaviour is defective prior to the new learning, the prediction depends on the relationship between the plasticity underlying the new behaviour and the plasticity underlying the defective old behaviour. The prediction has five parts.

**Figure 6:**
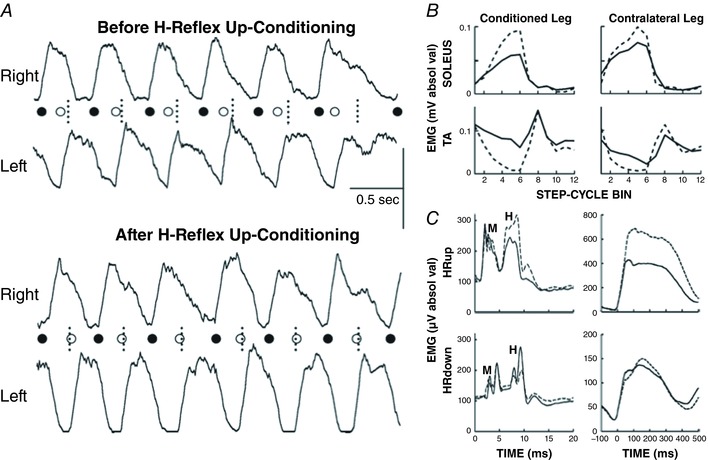
A new motor behaviour can improve an old behaviour after spinal cord injury in rats and humans *A*, the traces show the bursts of rectified electromyographic (EMG) activity from right and left soleus muscles during treadmill locomotion before (top) and after (bottom) up‐conditioning has increased the right soleus H‐reflex in a rat with a right lateral‐column (LC) transection. The onsets of the right and left soleus bursts (marked by the filled and open circles, respectively) reflect the onsets of the right and left stance phases, respectively. The short vertical dotted lines indicate the midpoints between right burst onsets, which is where the left burst onsets should occur. Before up‐conditioning, the left burst onset occurs too early, and the gait is asymmetrical (i.e. the rat limps). After up‐conditioning has strengthened the right soleus burst, the left burst onset occurs at the correct time, and the asymmetry is gone. Horizontal scale bar: 0.5 s; vertical scale bar: 100 and 150 μV for the right and left bursts, respectively. From Chen *et al*. (2006). *B*, rectified locomotor EMG activity in soleus and tibialis anterior (TA) muscles of both legs before (continuous line) and after (dashed line) unilateral soleus H‐reflex down‐conditioning in a person with spasticity due to incomplete spinal cord injury. The step cycle is divided into 12 equal bins, starting from foot contact. Thus, bins 1–7 are for the stance phase and bins 8–12 are for the swing phase. After successful down‐conditioning, EMG modulation over the step cycle is greater in the soleus and TA muscles of both legs. This is associated with a clinically significant increase in walking speed and improved right–left symmetry. From Thompson *et al*. (2013). *C*, effects of right H‐reflex up‐conditioning (HR_up_ rat) or down‐conditioning (HR_down_ rat) on soleus H‐reflexes and locomotor bursts in rats in which a right lateral column transection had produced limping due to weak right stance. The graphs show average post‐stimulus EMG activity in the conditioning protocol (left; M waves and H‐reflexes indicated) and average soleus locomotor bursts (right) in the control mode (continuous lines) and at the end of conditioning (dashed lines). Up‐conditioning increases the H‐reflex and the locomotor burst, and improves locomotion. Down‐conditioning decreases the H‐reflex, but it does not decrease the locomotor burst, and it does not further impair locomotion. See text for discussion. From Chen *et al*. (2014).

First, if the plasticity that produces the new behaviour also improves the old behaviour, the model predicts that its effect on the old behaviour will not be counteracted by compensatory plasticity: the old behaviour will get better. For example, if motoneuron excitation that is important for an old behaviour has been reduced by a spinal cord injury, new learning that strengthens motoneuron excitation should improve the old behaviour. This prediction was tested in rats in which transection or contusion of the right lateral column of the spinal cord had weakened the right stance phase of locomotion and produced asymmetrical stepping (i.e. the rats limped) (Chen *et al*. 2006, 2013). In these rats, up‐conditioning of the right soleus H‐reflex strengthened right stance and restored step symmetry. The stronger reflex pathway provided excitatory input to the motoneuron that substituted for input lost due to the injury; it thereby improved the old behaviour. Figure 6 *A* illustrates this result. In spinal cord‐injured rats that were not up‐conditioned, locomotion did not improve.

Second, if the spinal plasticity that produces a new behaviour also improves an old behaviour, the magnitude of this plasticity is likely to be enhanced because it will be driven by two of the participants in the negotiation, the new behaviour and the old. Human data support this prediction. In people with spasticity due to incomplete spinal cord injury, H‐reflex down‐conditioning can improve locomotion; moreover, the spinal contribution to the H‐reflex decrease is greater than it is in normal volunteers who undergo down‐conditioning (Thompson *et al*. 2013). For these individuals with spinal cord injury, spinal cord plasticity that decreases the H‐reflex is doubly adaptive: it benefits both the new behaviour (a smaller H‐reflex) and the old behaviour (locomotion). In contrast, for normal individuals, this plasticity benefits only the new behaviour; indeed, it may disturb the old behaviour (e.g. Fig. 5; unless its impact is counteracted by compensatory plasticity; Chen *et al*. 2011). Thus, the two behaviours oppose each other's influence on spinal properties; and the spinal contribution to the H‐reflex decrease is less pronounced (for discussion, Thompson *et al*. 2013).

Third, the model suggests that when a new behaviour produces plasticity that improves an old behaviour, it can lead to widespread plasticity that further improves the old behaviour. By producing the initial beneficial plasticity and by joining the ongoing negotiation among the behaviours, a new behaviour can move the state of the spinal cord to new territory in the multidimensional space defined by spinal neuronal and synaptic properties. Thus, it can enable the old behaviour to escape a suboptimal local minimum and achieve a more complete restoration of its key features. This prediction is supported by long‐term data from spinal cord‐injured rats like those described above (e.g. Fig. 6 *A*; Chen *et al*. 2006). After up‐conditioning ended, the H‐reflex pathway continued to grow stronger and locomotion continued to improve, even in the absence of continued up‐conditioning (Chen *et al*. 2014). Moreover, in humans in whom incomplete spinal cord injury had impaired locomotion by producing spasticity and foot‐drop, down‐conditioning of the soleus H‐reflex in the more impaired leg not only improved soleus locomotor activity in that leg, it also improved the locomotor activity of proximal and distal muscles in *both* legs (Thompson *et al*. 2013). This wider effect is illustrated in Fig. 6 *B*. People walked faster and more symmetrically, and they reported other improvements such as reduced fatigue and better balance. These gains went far beyond those attributable to the smaller soleus H‐reflex in the more impaired leg. They indicated much wider beneficial plasticity.

Fourth, if the plasticity that produces a new behaviour further impairs an old behaviour that is already impaired, the model predicts that compensatory plasticity will prevent further impairment: the key features of the old behaviour will not become even more defective. In rats in which contusion of the right lateral column of the spinal cord had weakened right stance and caused limping, down‐conditioning of the right soleus H‐reflex did not further weaken right stance (Chen *et al*. 2013). Even though the locomotor H‐reflex became smaller, the soleus locomotor burst was unchanged and limping did not worsen. It appeared that compensatory plasticity prevented further impairment of locomotion. Figure 6 *C* illustrates this finding.

Fifth, if the plasticity that produces a new behaviour further impairs an old behaviour, and the injury that impaired the old behaviour also disrupted the ascending pathways that provide the brain with performance information, the model predicts that compensatory plasticity will not occur and the new learning will further impair the old behaviour. This prediction remains to be tested.

In sum, studies of the impact of new learning on an impaired old behaviour support the picture of a negotiation among the new and old behaviours. When the new learning improves a pathway important to the old behaviour, it can trigger widespread changes that greatly improve the old behaviour. In contrast, when the new learning further impairs an important pathway, it leads to compensatory plasticity that prevents further impairment of the old behaviour.

## Practical and theoretical implications of the model

### Targeted neuroplasticity for rehabilitation

As described above, in both rats and humans with incomplete spinal cord injuries, operantly conditioned change in a relevant reflex pathway improved locomotion (Chen *et al*. 2006, 2013; Manella *et al*. 2013; Thompson *et al*. 2013). This improvement was achieved long after the injury, when spontaneous recovery (in the rats) and standard rehabilitation regimens (in the humans) had already exerted their beneficial effects and a stable deficit remained. Why did the improvement occur? Or rather, why had it not occurred previously, since the capacity for beneficial change in the H‐reflex pathway was certainly present prior to application of the conditioning protocol?

The negotiated equilibrium model provides three possible answers to this question, any or all of which may be correct. First, the spinal cord injury could have impaired or abolished the ascending sensory input that would otherwise have guided appropriate spinal and supraspinal changes, including beneficial change in the H‐reflex pathway. By replacing the lost ascending sensory input with the reward contingency, the conditioning protocol guided the beneficial change in the reflex pathway. The data in Fig. 5 support this possibility.

Second, the spinal cord injury might have affected the substrate of brain and spinal plasticity underlying another important behaviour (e.g. postural maintenance or flexion withdrawal), so that the ongoing negotiation among the behaviours resulted in an H‐reflex pathway less satisfactory for locomotion. By adding an appropriately selected new behaviour to the negotiation, the conditioning protocol shifted the balance in favor of locomotion.

Third, in the context of the ongoing negotiation occurring in the damaged CNS, the change in CST activity that produces the beneficial plasticity in the H‐reflex pathway might not have been sufficiently rewarding to be induced and maintained. In order to be operantly conditioned, that is, to be induced and maintained, this change in CST activity needs to be consistently rewarding for an important behaviour (e.g. locomotion) in a setting in which many other changes in activity are also occurring throughout the CNS. If these other changes have greater immediate impacts on locomotion, CST activity may not change appropriately. A modelling study of stroke recovery (Reinkensmeyer *et al*. 2012) suggests why this is so. If changes in many different kinds of activity can improve a behaviour, the changes that have greater impact will occur, while those with less impact may not occur at all. The H‐reflex conditioning protocol adds to the negotiation a new behaviour for which the change in CST activity does have major impact. The protocol ensures that the change in CST activity is consistently rewarding and is therefore maintained. Locomotion is a collateral beneficiary; it too is improved by the resulting change in the H‐reflex pathway. Furthermore, as discussed above, the change in the H‐reflex pathway may escape an inferior local minimum in the multidimensional space defined by all spinal properties, and it may thereby lead to widespread beneficial plasticity.

The importance of each of these possibilities in producing the benefits of interventions that target plasticity to particular sites is likely to vary across CNS disorders, behavioural impairments and individuals. The negotiated equilibrium model can help to guide the development and evaluation of these interventions; and they in turn can test and elaborate the model. By complementing standard rehabilitation methods, these interventions should be able to enhance recovery of locomotion and other important behaviours. Furthermore, once a targeted intervention such as H‐reflex conditioning has triggered widespread beneficial plasticity, the benefits may become self‐sustaining so that continued exposure to the intervention is no longer needed (Chen *et al*. 2014).

### Negotiated equilibria elsewhere in the CNS?

The negotiated equilibrium model was developed for and is most clearly applicable to the spinal cord. It reconciles the continual plasticity that occurs in the spinal cord with its reliability as the final common pathway for motor behaviours. At the same time, the spinal cord is not the only CNS region that serves many behaviours. In primates particularly, activity in sensorimotor cortical areas underlies motor skills. As new behaviours are acquired or when peripheral or central lesions occur, the cortex changes on macro (i.e. regional) and micro (i.e. neuronal and synaptic) levels, and the relationships between cortical activity and movement are affected (reviewed in Adkins *et al*. 2006; Francis & Song, 2011; Sur *et al*. 2013). Nevertheless, the CNS continues to reliably support a large repertoire of behaviours. This reliability may reflect cortical interaction among the behaviours similar to that occurring in the spinal cord. As with spinal cord activity, the cortical activity underlying a behaviour may change, but the key features of the behaviour are maintained.

**Figure 7:**
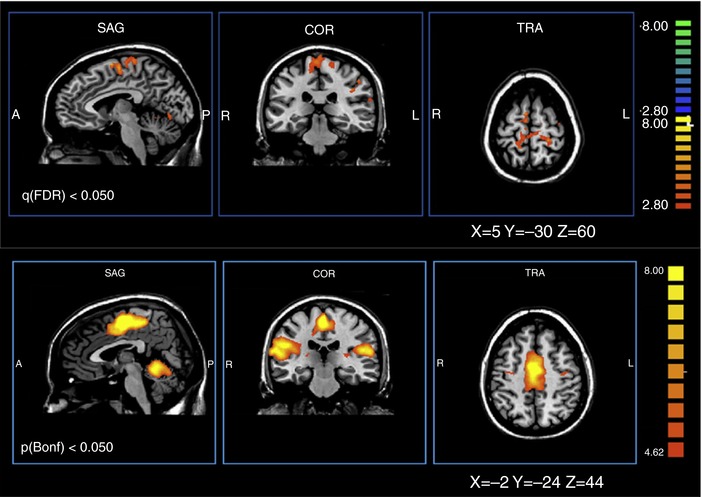
Evidence that negotiated equilibria may exist in cortex Dance training changes the cortical activity underlying a simple foot flexion/extension behaviour. Top: areas of differential activation: post‐training condition minus pre‐training condition. Orthogonal views. Bottom: areas of differential connectivity: post‐training condition minus pre‐training condition. From Sacco *et al*. (2009), Elsevier, with permission.

An interesting fMRI study is consistent with this possibility (Sacco *et al*. 2009). After normal adults mastered a simple foot flexion/extension behaviour, fMRI was used to assess the cortical activity underlying this performance. The subjects then participated in a dance class over 2 weeks; and the fMRI assessment of the simple flexion/extension behaviour was repeated. Fig. 7 shows the cortical areas in which the activity or the interareal connectivity associated with the simple behaviour was different after the several weeks of training on another behaviour (i.e. dancing) that also involved the leg. The simple behaviour was still performed satisfactorily, but the CNS activity underlying it had changed substantially. Current studies using brain–computer interface technology to explore the interactions among the patterns of cortical activity underlying different virtual behaviours may provide further insight into the wider applicability of the negotiated equilibrium model (e.g. Sadtler *et al*. 2014).

In a recent modelling study, Ajemian *et al*. (2013) addressed the question of how the CNS, and the cortex in particular, can continually acquire new behaviours while preserving old behaviours. Beginning from the inadequacy of the traditional notion of fixed memory traces, their analysis shows that a neural network composed of individual elements (e.g. synapses) that are hyperplastic, very noisy and massively redundant could acquire and maintain multiple behaviours at rates and with reliability consistent with observations of standard motor behaviours. The authors support this model with existing data on the modification and retention of dendritic spines in cortex. The model describes an adaptive process through which behaviours concurrently create and maintain patterns of plasticity that ensure their performances, a process similar to what the negotiated equilibrium model envisions in the spinal cord.

At the same time, the models differ in important ways. The Ajemian *et al*. (2013) model posits a very high degree of redundancy, which may well be present in cortex and other brain areas. Such redundancy is clearly not present in the spinal cord, where many behaviours must necessarily share the same limited numbers of motoneurons and interneurons and the same synaptic connections. Furthermore, the hyperplasticity of the Ajemian *et al*. (2013) cortical model does not appear to be consistent with the functional reliability of the normal spinal cord. If just a few repetitions of a behaviour could markedly change the highly shared elements of the spinal cord, the next behaviour would encounter a final common pathway with new properties, and would require its own repetitions to re‐establish satisfactory performance; such rapid plasticity would prevent the spinal cord from being a reliable final common pathway for all behaviours. Instead, spinal cord plasticity appears to be a gradual process with similarly gradual functional consequences (illustrated by the slow adaptive changes in H‐reflex size shown in Fig. 2). The process is likely to be guided by CST and other descending activity that reflects the patterns of brain plasticity underlying the current repertoire of behaviours. This influence ensures a reliable final common pathway for the entire repertoire.

### The CNS as a multi‐user system

The newly appreciated role of spinal cord plasticity in motor learning and recent explorations of how that plasticity relates to plasticity in the brain (see above) indicate that the CNS substrate of a motor behaviour is not limited to the cerebrum. Motor behaviours entail plasticity that is distributed from the cortex to the spinal cord. Their distributed substrates may operate as hierarchies. How the cerebral components of motor behaviours responsible for different behaviours interact with each other in the brain is largely unknown. Nevertheless, it is clear that they come together in the spinal cord, where they must share interneurons and control muscles through the same small numbers of motoneurons. The negotiated equilibrium model is an effort to explain how this overlap functions, how it comes about that all the behaviours in the repertoire are satisfactorily maintained.

According to the negotiated equilibrium model, the CNS is inhabited by a repertoire of numerous behaviours. The substrate of each motor behaviour is a network of brain and spinal cord plasticity that functions as a hierarchy: guided by performance information, the plasticity in the brain induces and maintains the plasticity in the spinal cord. Each behaviour operates as an independent agent; it is continually inducing plasticity to maintain its key features despite plasticity induced by other behaviours and by growth, ageing, trauma and disease, and other life events. This concept recalls Bernstein's (1967) assertion that motor behaviours (e.g. locomotion), which he refers to as *biodynamic structures*, ‘live and develop’. Motor behaviours inevitably overlap in the spinal cord and it is there that they interact most obviously and intensively. Their interactions can be cooperative, compensatory or competitive. Two behaviours might both increase the firing threshold of a neuron (i.e. cooperation), one might increase the threshold while another strengthens excitatory synaptic input to the neuron (i.e. compensation), or one might increase the threshold while another decreases it (i.e. competition). For example, when ballet training reduces primary afferent impact on spinal motoneurons (thereby facilitating muscle coactivation), locomotion may strengthen other excitatory inputs (thereby preserving the stance‐phase burst).

These complex interactions among multiple independent agents invite analysis in terms of game theory (Davis, 1983; Gibbons, 1992; Fudenberg & Tirole, 2005). The motor behaviours that share the spinal cord are all players in the game, and the payoff for each is the degree to which its own key features are achieved and maintained from moment to moment and year to year. At the same time, assuming that every behaviour is important to the well‐being of the individual, an effective negotiation is one in which every behaviour consistently receives an acceptable payoff. The deleterious impact of having a single behaviour dominate a negotiated equilibrium is illustrated by the problems caused by clonus in people with spinal cord injuries, and also (if negotiated equilibria are present in the brain) by the often devastating consequences of addictive or obsessive–compulsive behaviours.

The heuristic value of approaching interactions among behaviours in terms of game theory will depend on the extent to which this approach can explain the plasticity produced by these interactions and how this plasticity enables sensorimotor function. For example, it is generally assumed that there is a single basic pattern of neuronal activity for each motor behaviour, and that individual performances of the behaviour (i.e. trials) only diverge from this pattern due to factors extraneous to the behaviour itself. That is, it is assumed that the CNS has a single strategy, or programme, for producing a given behaviour. However, if the feedback associated with each performance of a behaviour induces plasticity aimed at optimizing its key features, and other behaviours are concurrently inducing plasticity at many of the same sites, game theory suggests that a behaviour might best ensure its own consistently good performance with a mixed strategy, in which each of several different patterns of activity produces a certain proportion of the performances (Davis, 1983; Gibbons, 1992; Fudenberg & Tirole, 2005). That is, an appropriate mix of strategies might ensure a negotiated equilibrium most beneficial to the behaviour. This possibility has yet to be explored; its confirmation could profoundly affect understanding of motor learning and motor performance.

### Relations to other models of CNS function

Most models of CNS function propose principles or processes that apply to any individual behaviour (e.g. Friston 2010) or they endeavor to explain how a particular kind of behaviour is produced (e.g. Shadmehr *et* *al*. 2010; Ingram *et al*. 2017). Among the latter, the equilibrium point hypothesis (Feldman 1966; Sainburg 2015) in particular warrants mention in order to avoid any potential confusion with the negotiated equilibrium model presented in this paper. In brief, the equilibrium point hypothesis addresses the question of how movement is initiated and controlled; it proposes that the CNS produces limb movements (or maintains limb position against changing external forces) by modifying the threshold lengths at which the muscles controlling the limb become active.

In contrast to such models of CNS function, the negotiated equilibrium model addresses a very specific practical problem: it attempts to explain how a continually changing spinal cord manages to provide a reliable final common pathway for the many behaviours that must share it. As presented here, the model does this in a substantive evidence‐based fashion for the spinal cord; and the presentation extends the explanation in a preliminary and highly speculative fashion to the brain in general. The model's most distinctive feature is the proposition that the substrates of behaviours are independent agents that establish and maintain themselves in the CNS.

This proposition is compatible with current models of how the CNS produces individual motor behaviours (e.g. Ingram *et al*. 2017). And, assuming that these independent agents also accommodate each other for the overall benefit of the organism, the proposition is compatible with models that specify the terms of this accommodation (e.g. Friston, 2010). In sum, the contribution of the negotiated equilibrium model is new insight into how multiple behaviours may share the limited resources of the spinal cord, and perhaps those of the brain as well.

### Further studies

At present, much of the evidence for the negotiated equilibrium model comes from animal and human studies of spinal reflex conditioning and its interactions with locomotion. The wider applicability of the model depends on the extent to which it is able to account for the interactions among other motor behaviours, is consistent with the underlying changes in spinal neurons and synapses, and can explain the relationships between the cerebral and spinal components of the plasticity responsible for these behaviours.

Studies testing the wider validity of the model can benefit from the relative simplicity of the spinal cord, its direct mapping to behaviour, and its connection to the brain through well‐defined pathways – the same advantages that have made the spinal cord a uniquely valuable experimental system for centuries. Furthermore, animal and human studies can contribute in complementary ways. Animal studies can impose completely characterized motor learning experiences (or even life‐long sequences of such experiences) and can employ precise interventions (e.g. reversible or irreversible lesions, optogenetic stimulation); they can thereby explore mechanisms on the systems level as well as on neuronal and synaptic levels. Human studies can engage more varied experiences and intricate behaviours; they may benefit from the subjects’ verbal reports; and they can take advantage of non‐invasive imaging and stimulation methods and the extensive cortical areas of humans to explore the cerebrospinal interactions that create and maintain behaviours.

In testing the model, a variety of questions warrant study. One is whether the model can account for the development, maintenance and modification of muscle synergies, the stereotyped combinations of muscle activations that appear to be the building blocks of complex motor behaviours (Bizzi *et al*. 2008; Berger *et al*. 2013; Sawers *et al*. 2015). These synergies may be the end products of long‐term negotiation among established behaviours.

At present, the principal evidence for negotiation among behaviours consists of long‐term data, such as the changes in locomotor EMG activity and kinematics after 50 days of H‐reflex conditioning (Chen *et al*. 2005, 2011). Thus, a second important question is whether the model can account for short‐term (moment‐to‐moment or day‐to‐day) effects of one behaviour on another. It would also be worthwhile to explore the effects of interventions, such as contingent vagal nerve stimulation (Hays *et al*. 2013), that can enhance a particular behaviour. The model predicts that such an intervention, by strengthening the influence of that behaviour in the negotiation among behaviours, would affect, and perhaps even impair, other behaviours.

Another question is the model's relation to the phenomenon of structure (or structural) learning, in which a new behaviour similar to one or more old behaviours can be acquired relatively quickly (e.g. tennis, badminton, ping pong) (Braun *et al*. 2009, 2010). In terms of the model, structural learning may be understandable as the combining of the old and new behaviours into a single behaviour, the substrate of which can produce either the old or new behaviour and can select between them as needed. In game theory terms, the combination of the new and old behaviours could be understood as a mutually beneficial alliance between two players. The resulting synergism could provide them with greater influence in the ongoing negotiation with other behaviours that shapes the neuronal and synaptic properties on which they all depend.

A particularly important question is whether negotiation occurs continually, even without actual behaviours. The fact that some behaviours occur infrequently, yet are preserved despite new learning, suggests that the plasticity underlying their preservation does not result only from their few occurrences. The growing evidence that ongoing overtly spontaneous brain activity contributes to learning is consistent with the possibility of continual negotiation (e.g. Albert *et al*. 2009; Litwin‐Kumar & Doiron, 2014). Whether this activity can be parsed into ongoing negotiation among behaviours and, if so, how guidance is provided in the absence of behaviour, remain to be determined.

Further exploration of potential therapeutic applications of the model is particularly appealing, both theoretically and practically. If the model is widely applicable, it should be able to: guide rehabilitation of motor functions other than locomotion; predict the long‐term results of specific interventions, whether they are new motor learning experiences or pathway‐specific stimulation regimens; and elucidate inter‐individual variability in results in terms of differences in past motor learning. To the extent they are successful, these studies could further establish the model and contribute to the realization of new therapeutic methods.

Whatever their implications for the negotiated equilibrium model, the studies addressing these questions and related issues should improve, and may substantially alter, understanding of how the CNS interacts effectively with the world. As the principal interface for these interactions, the spinal cord has an essential role. When it was assumed to be hardwired, its role appeared to be quite simple; with recent recognition of its ongoing plasticity, this is no longer the case. It is now clear that effective efforts to explain the acquisition and lifelong maintenance of motor behaviours cannot focus entirely on the brain; they must incorporate and account for spinal cord plasticity. Furthermore, they must recognize and accommodate the interactions among behaviours that surely occur in the spinal cord and perhaps in the brain as well.

## Additional information

### Competing interests

None.

### Funding

Work in the author's laboratory has been supported by NIH grants NS22189, HD36020, NS061823, HD32571 and 1P41EB018783, VA Merit Award 1 I01 BX002550, the United Cerebral Palsy Research and Educational Foundation, the International Spinal Research Trust, the Paralyzed Veterans of America, the Christopher and Dana Reeve Paralysis Foundation, and the New York State Spinal Cord Injury Research Board.

## References

[tjp12961-bib-0001] Adkins DL , Boychuk J , Remple MS & Kleim JA (2006). Motor training induces experience‐specific patterns of plasticity across motor cortex and spinal cord. J Appl Physiol 101, 1776–1782. 16959909 10.1152/japplphysiol.00515.2006

[tjp12961-bib-0002] Ajemian R , D'Ausilio A , Moormand H & Bizzi E (2013). A theory for how sensorimotor skills are learned and retained in noisy and nonstationary neural circuits. Proc Natl Acad Sci U S A 110, E5078–E5087. 24324147 10.1073/pnas.1320116110 PMC3876265

[tjp12961-bib-0003] Albert NB , Robertson EM , Mehta P & Miall RC (2009). Resting state networks and memory consolidation. Comm Integ Biol 2, 530–532. 10.4161/cib.2.6.9612 PMC2829828 20195459

[tjp12961-bib-0004] Baldissera F , Hultborn H & Illert M (1981). Integration in spinal neuronal systems In *Handbook of Physiology*, Section I, *The Nervous System*, Vol. II, *Motor Control*, Part I, ed. Brooks VB, pp. 509–595. Williams and Wilkins, Baltimore.

[tjp12961-bib-0005] Barbeau H & Rossignol S (1987). Recovery of locomotion after chronic spinalization in the adult cat. Brain Res 412, 84–95. 3607464 10.1016/0006-8993(87)91442-9

[tjp12961-bib-0006] Becker D & McDonald JW 3rd (2012). Approaches to repairing the damaged spinal cord: overview. Handb Clin Neurol 109, 445–461. 23098730 10.1016/B978-0-444-52137-8.00028-0

[tjp12961-bib-0007] Berger DJ , Gentner R , Edmunds T , Pai DK & D'Avella A (2013). Differences in adaptation rates after virtual surgeries provide direct evidence for modularity. J Neurosci 33, 12384–12394. 23884944 10.1523/JNEUROSCI.0122-13.2013 PMC6618678

[tjp12961-bib-0008] Bernstein NA (1967). The Co‐ordination and Regulation of Movements. Pergamon Press, Oxford.

[tjp12961-bib-0009] Bizzi E , Cheung VC , D'Avella A , Saltiel P & Tresch M (2008). Combining modules for movement. Brain Res Rev 57, 125–133. 18029291 10.1016/j.brainresrev.2007.08.004 PMC4295773

[tjp12961-bib-0010] Boyden ES , Katoh A & Raymond JL (2004). Cerebellum‐dependent learning: the role of multiple plasticity mechanisms. Annu Rev Neurosci 27, 581–609. 15217344 10.1146/annurev.neuro.27.070203.144238

[tjp12961-bib-0011] Braun DA , Aertsen A , Wolpert DM & Mehring C (2009). Motor task variation induces structural learning. Curr Biol 19, 352–357. 19217296 10.1016/j.cub.2009.01.036 PMC2669412

[tjp12961-bib-0012] Braun DA , Waldert S , Aertsen A , Wolpert DM & Mehring C (2010). Structure learning in a sensorimotor association task. PLoS One 5, e8973. 20126409 10.1371/journal.pone.0008973 PMC2813299

[tjp12961-bib-0013] Brodal A (1981). Neurological Anatomy in Relation to Clinical Medicine. Oxford University Press, New York.

[tjp12961-bib-0014] Brown WF (1984). The Physiological and Technical Basis of Electromyography. Butterworths, Boston.

[tjp12961-bib-0015] Cardenas DD & Hooton TM (1995). Urinary tract infection in persons with spinal cord injury. Arch Phys Med Rehabil 76, 272–280. 7717822 10.1016/s0003-9993(95)80615-6

[tjp12961-bib-0016] Carmel JB , Berrol LJ , Brus‐Ramer M & Martin JH (2010). Chronic electrical stimulation of the intact corticospinal system after unilateral injury restores skilled locomotor control and promotes spinal axon outgrowth. J Neurosci 30, 10918–10926. 20702720 10.1523/JNEUROSCI.1435-10.2010 PMC2929360

[tjp12961-bib-0017] Carp JS , Tennissen AM , Chen XY & Wolpaw JR (2006). H‐reflex operant conditioning in mice. J Neurophysiol 96, 1718–1727. 16837659 10.1152/jn.00470.2006

[tjp12961-bib-0018] Carrier L , Brustein E & Rossignol S (1997). Locomotion of the hindlimbs after neurectomy of ankle flexors in intact and spinal cats: model for the study of locomotor plasticity. J Neurophysiol 77, 1979–1993. 9114249 10.1152/jn.1997.77.4.1979

[tjp12961-bib-0019] Ceccato J‐C , de Sèze M , Azevedo C & Cazalets J‐R (2009). Comparison of trunk activity during gait initiation and walking in humans. PLoS One 4, e8193. 19997606 10.1371/journal.pone.0008193 PMC2782139

[tjp12961-bib-0020] Chamberlain T , Halick P & Gerard RW (1963). Fixation of experience in the rat spinal cord. J Neurophysiol 22, 662–673. 10.1152/jn.1963.26.4.662 14019944

[tjp12961-bib-0021] Chang Y‐H , Auyang AG , Scholz JP & Nichols TR (2009). Whole limb kinematics are preferentially conserved over individual joint kinematics after peripheral nerve injury. J Exp Biol 212, 3511–3521. 19837893 10.1242/jeb.033886 PMC2762878

[tjp12961-bib-0022] Chen XY , Carp JS , Chen L & Wolpaw JR (2002). Corticospinal tract transection prevents operantly conditioned increase of H‐reflex in rats. Exp Brain Res 144, 88–94. 11976762 10.1007/s00221-002-1026-8

[tjp12961-bib-0023] Chen XY , Chen L & Wolpaw JR (2003). Conditioned H‐reflex increase persists after transection of the main corticospinal tract in rats. J Neurophysiol 90, 3572–3578. 12917382 10.1152/jn.00264.2003

[tjp12961-bib-0024] Chen XY , Feng‐Chen KC , Chen L , Stark DM & Wolpaw JR (2001). Short‐term and medium‐term effects of spinal cord tract transections on soleus H‐reflex in freely moving rats. J Neurotrama 18, 313–327. 10.1089/08977150151070973 11284551

[tjp12961-bib-0025] Chen XY , Wang Y , Chen Y , Chen L & Wolpaw JR (2016a). Ablation of the inferior olive prevents H‐reflex down‐conditioning in rats. J Neurophysiol 115, 1630–1636. 26792888 10.1152/jn.01069.2015 PMC4808093

[tjp12961-bib-0026] Chen XY , Wang Y , Chen Y , Chen L & Wolpaw JR (2016b). The inferior olive is essential for long‐term maintenance of a simple motor skill. J Neurophysiol 116, 1946–1955. 27535367 10.1152/jn.00085.2016 PMC5144694

[tjp12961-bib-0027] Chen XY & Wolpaw JR (1995). Operant conditioning of H‐reflex in freely moving rats. J Neurophysiol 73, 411–415. 7714584 10.1152/jn.1995.73.1.411

[tjp12961-bib-0028] Chen XY & Wolpaw JR (1997). Dorsal column but not lateral column transection prevents down conditioning of H‐reflex in rats. J Neurophysiol 78, 1730–1734. 9310458 10.1152/jn.1997.78.3.1730

[tjp12961-bib-0029] Chen XY & Wolpaw JR (2002). Probable corticospinal tract control of spinal cord plasticity in rats. J Neurophysiol 87, 645–652. 11826033 10.1152/jn.00391.2001

[tjp12961-bib-0030] Chen XY & Wolpaw JR (2005). Ablation of cerebellar nuclei prevents H‐reflex down‐conditioning in rats. Learn Mem 12, 248–254. 15930503 10.1101/lm.91305 PMC1142452

[tjp12961-bib-0031] Chen Y , Chen L , Liu RL , Wang Y , Chen XY & Wolpaw JR (2013). Locomotor impact of beneficial or non‐beneficial H‐reflex conditioning after spinal cord injury. J Neurophysiol 111, 1249–1258. 24371288 10.1152/jn.00756.2013 PMC3949309

[tjp12961-bib-0032] Chen Y , Chen L , Wang Y , Chen XY & Wolpaw JR (2017). Why new spinal cord plasticity does not disrupt old motor behaviors. J Neurosci 37, 8198–8206. 28743726 10.1523/JNEUROSCI.0767-17.2017 PMC5566867

[tjp12961-bib-0033] Chen Y , Chen L , Wang Y , Wolpaw JR & Chen XY (2011). Operant conditioning of rat soleus H‐reflex oppositely affects another H‐reflex and changes locomotor kinematics. J Neurosci 31, 11370–11375. 21813696 10.1523/JNEUROSCI.1526-11.2011 PMC3156437

[tjp12961-bib-0034] Chen Y , Chen L , Wang Y , Wolpaw JR & Chen XY (2014). Persistent beneficial impact of H‐reflex conditioning in spinal cord injured rats. J Neurophysiol 112, 2374–2381. 25143542 10.1152/jn.00422.2014 PMC4233264

[tjp12961-bib-0035] Chen Y , Chen XY , Jakeman LB , Chen L , Stokes BT & Wolpaw JR (2006). Operant conditioning of H‐reflex can correct a locomotor abnormality after spinal cord injury in rats. J Neurosci 26, 12537–12543. 17135415 10.1523/JNEUROSCI.2198-06.2006 PMC6674902

[tjp12961-bib-0036] Chen Y , Chen XY , Jakeman LB , Schalk G , Stokes BT & Wolpaw JR (2005). The interaction of a new motor skill and an old one: H‐reflex conditioning and locomotion in rats. J Neurosci 25, 6898–6906. 16033899 10.1523/JNEUROSCI.1684-05.2005 PMC6725342

[tjp12961-bib-0037] Cheron G , Dan B & Márquez‐Ruiz J (2013). Translational approach to behavioral learning: Lessons from cerebellar plasticity. Neur Plast 2013, 853654. 10.1155/2013/853654 PMC3844268 24319600

[tjp12961-bib-0038] Christiansen L , Lundbye‐Jensen J , Perez MA & Nielsen JB (2017). How plastic are human spinal cord motor circuitries? Exp Brain Res 235, 3243–3249. 28776155 10.1007/s00221-017-5037-x

[tjp12961-bib-0039] Clarke E & Jacyna LS (1987). Nineteenth‐Century Origins of Neuroscientific Concepts. Unversity of California Press, Berkeley. 10.1017/s002572730004802x PMC1139860 3287061

[tjp12961-bib-0040] Clarke E & O'Malley CD (1996). The Human Brain and Spinal Cord. Norman, San Francisco.

[tjp12961-bib-0041] (1993). Compact Oxford English Dictionary, 2nd edn, eds Weiner ESC & Simpson JA, p. 1782 Oxford University Press, Oxford.

[tjp12961-bib-0042] Courtine G , Gerasimenko Y , Van Den Brand R , Yew A , Musienko P , Zhong H , Song B , Ao Y , Ichiyama RM , Lavrov I , Roy RR , Sofroniew MV & Edgerton VR (2009). Transformation of nonfunctional spinal circuits into functional states after the loss of brain input. Nat Neurosci 12, 1333–1342. 19767747 10.1038/nn.2401 PMC2828944

[tjp12961-bib-0043] Cregg JM , DePaul MA , Filous AR , Lang BT , Tran A & Silver J (2014).Functional regeneration beyond the glial scar. Exp Neurol 253, 197–207. 24424280 10.1016/j.expneurol.2013.12.024 PMC3951813

[tjp12961-bib-0044] Davis MD (1983). Game Theory: A Nontechnical Introduction. Mineola, Dover.

[tjp12961-bib-0045] DiGiorgio AM (1929). Persistence of postural and motor asymmetries of cerebellar origin in spinal animals: I, II, III. Arch Fisio 27, 518–580.

[tjp12961-bib-0046] DiGiorgio AM (1942). Influences of the cerebellum‐neocerebellum on the postural tone of the limbs and cerebellar somatotopy in the rhomboencephalic animal. Arch Fisiol 42, 25–79.

[tjp12961-bib-0047] Dragert K & Zehr EP (2011). Bilateral neuromuscular plasticity from unilateral training of the ankle dorsiflexors. Exp Brain Res 208, 217–227. 21069308 10.1007/s00221-010-2472-3

[tjp12961-bib-0048] Durkovic RG & Damianopoulos EN (1986). Forward and backward classical conditioning of the flexion reflex in the spinal cat. J Neurosci 6, 2921–2925. 3760942 10.1523/JNEUROSCI.06-10-02921.1986 PMC6568774

[tjp12961-bib-0049] Edgerton VR & Roy RR (2009). Activity‐dependent plasticity of spinal locomotion: implications for sensory processing. Exerc Sport Sci Rev 37, 171–178. 19955866 10.1097/JES.0b013e3181b7b932 PMC2790155

[tjp12961-bib-0050] Evatt ML , Wolf SL & Segal RL (1989). Modification of human spinal stretch reflexes: preliminary studies. Neurosci Lett 105, 350–355. 2594221 10.1016/0304-3940(89)90646-0

[tjp12961-bib-0051] Eyre JA , Taylor JP , Villagra F , Smith M & Miller S (2001). Evidence of activity‐dependent withdrawal of corticospinal projections during human development. Neurology 57, 1543–1554. 11706088 10.1212/wnl.57.9.1543

[tjp12961-bib-0052] Feldman AG (1966). Functional tuning of the nervous system with control of movement or maintenance of a steady posture, II. Controllable parameters of the muscles. Biophysics 11, 565–578.

[tjp12961-bib-0053] Filli L , Engmann AK , Zörner B , Weinmann O , Moraitis T , Gullo M , Kasper H , Schneider R & Schwab ME (2014). Bridging the gap: a reticulo‐propriospinal detour bypassing an incomplete spinal cord injury. J Neurosci 34, 13399–13410. 25274818 10.1523/JNEUROSCI.0701-14.2014 PMC6608315

[tjp12961-bib-0054] Fouad K , Krajacica A & Tetzlaff W (2011). Spinal cord injury and plasticity: opportunities and challenges. Brain Res Bull 84, 337–342. 20471456 10.1016/j.brainresbull.2010.04.017

[tjp12961-bib-0055] Francis JT & Song W (2011). Neuroplasticity of the sensorimotor cortex during learning. Neural Plast 2011, 310737. 21949908 10.1155/2011/310737 PMC3178113

[tjp12961-bib-0056] Freeman JH & Steinmetz AB (2011). Neural circuitry and plasticity mechanisms underlying delay eyeblink conditioning. Learn Mem 18, 666–677. 21969489 10.1101/lm.2023011 PMC3861981

[tjp12961-bib-0057] Frigon A & Rossignol S (2009). Partial denervation of ankle extensors prior to spinalization in cats impacts the expression of locomotion and the phasic modulation of reflexes. Neuroscience 158, 1675–1690. 19056469 10.1016/j.neuroscience.2008.11.005

[tjp12961-bib-0058] Friston K (2010). The free energy principle: a unified brain theory? Nat Rev Neurosci 11, 127–138. 20068583 10.1038/nrn2787

[tjp12961-bib-0059] Fudenberg D & Tirole J (2005). Game Theory. ANE Books, New Delhi.

[tjp12961-bib-0060] Geertsen SS , Lundbye‐Jensen J & Nielsen JB (2008). Increased central facilitation of antagonist reciprocal inhibition at the onset of dorsiflexion following explosive strength training. J App Physiol 105, 915–922. 10.1152/japplphysiol.01155.2007 18583382

[tjp12961-bib-0061] Gibbons R (1992). Game Theory for Applied Economists. Princeton University Press, Princeton.

[tjp12961-bib-0062] Grau JW (2013). Learning from the spinal cord: How the study of spinal cord plasticity informs our view of learning. Neurobiol Learn Mem 108C, 155–171. 10.1016/j.nlm.2013.08.003 PMC3946174 23973905

[tjp12961-bib-0063] Harkema SJ , Hillyer J , Schmidt‐Read M , Ardolino E , Sisto SA & Behrman AL (2012).Locomotor training: As a treatment of spinal cord injury and in the progression of neurologic rehabilitation. Arch Phys Med Rehabil 93, 1588–1597. 22920456 10.1016/j.apmr.2012.04.032

[tjp12961-bib-0064] Hays SA , Rennaker RL & Kilgard MP (2013).Targeting plasticity with vagus nerve stimulation to treat neurological disease. Prog Brain Res 207, 275–299. 24309259 10.1016/B978-0-444-63327-9.00010-2 PMC4615598

[tjp12961-bib-0065] Henneman E , Mendell LM (1981).Functional organization of motoneurons pool and inputs In *Handbook of Physiology*, Section 1, *The Nervous System*, Vol. 2, *Motor Control*, ed. Brooks VB, pp. 423–507. American Physiological Society, Bethesda.

[tjp12961-bib-0066] Henzel MK , Bogie KM , Guihan M & Ho CH (2011). Pressure ulcer management and research priorities for patients with spinal cord injury: consensus opinion from SCI QUERI Expert Panel on Pressure Ulcer Research Implementation. J Rehabil Res Dev 48, xi–xxxii. 10.1682/jrrd.2011.01.0011 21480093

[tjp12961-bib-0067] Hiersemenzel LP , Curt A & Dietz V (2000). From spinal shock to spasticity: neuronal adaptations to a spinal cord injury. Neurology 54, 1574–1582. 10762496 10.1212/wnl.54.8.1574

[tjp12961-bib-0068] Horridge G (1962). Learning of leg position by headless insects. Nature 193, 697–698. 14449018 10.1038/193697a0

[tjp12961-bib-0069] Ingram JN , Sadeghi M , Flanagan JR & Wolpert DM (2017). An error‐tuned model for sensorimotor learning. PLoS Comput Biol 13, e1005883. 29253869 10.1371/journal.pcbi.1005883 PMC5749863

[tjp12961-bib-0070] Juknis N , Cooper JM & Volshteyn O (2012). The changing landscape of spinal cord injury. Handb Clin Neurol 109, 149–166. 23098711 10.1016/B978-0-444-52137-8.00009-7

[tjp12961-bib-0071] Kido A , Tanaka N & Stein RB (2004). Spinal excitation and inhibition in humans at different ages. Can J Physiol Pharmacol 82, 238–248. 15181462 10.1139/y04-017

[tjp12961-bib-0072] Kilgannon C (1996). By their walk shall you know them. *New York Times*, November 10, 1996, http://www.nytimes.com/1996/11/10/nyregion/by-their-walk-shall-you-know-them.html.

[tjp12961-bib-0073] Koceja DM , Markus CA & Trimble MH (1995). Postural modulation of the soleus H reflex in young and old subjects. Electroencephalogr Clin Neurophysiol 97, 387–393. 8536590 10.1016/0924-980x(95)00163-f

[tjp12961-bib-0074] Latash ML , Scholz JP & Schöner G (2007). Toward a new theory of motor synergies. Mot Control 11, 275–307. 10.1123/mcj.11.3.276 17715460

[tjp12961-bib-0075] Leergaard TB , Lillehaug S , Schutter ED , Bower JM & Bjaalie JG (2006). Topographical organization of pathways from somatosensory cortex through the pontine nuclei to tactile regions of the rat cerebellar hemispheres. Eur J Neurosci 24, 2801–2812. 17156205 10.1111/j.1460-9568.2006.05150.x

[tjp12961-bib-0076] Levinsson A , Luo XL , Holmberg H & Schouenborg J (1999). Developmental tuning in a spinal nociceptive system: effects of neonatal spinalization. J Neurosci 19, 10397–10403. 10575037 10.1523/JNEUROSCI.19-23-10397.1999 PMC6782439

[tjp12961-bib-0077] Liddell EGT (1960). The Discovery of Reflexes. Clarendon Press, Oxford.

[tjp12961-bib-0078] Litwin‐Kumar A & Doiron B (2014). Formation and maintenance of neuronal assemblies through synaptic plasticity. Nature Commun 5, 5319. 25395015 10.1038/ncomms6319

[tjp12961-bib-0079] Liu S , Schackel T , Weidner N & Puttagunta R (2018). Biomaterial‐supported cell transplantation treatments for spinal cord injury: challenges and perspectives. Front Cell Neurosci 11, 430. 29375316 10.3389/fncel.2017.00430 PMC5768640

[tjp12961-bib-0080] Longley M & Yeo CH (2014). Distribution of neural plasticity in cerebellum‐dependent motor learning. Prog Brain Res 210, 79–101. 24916290 10.1016/B978-0-444-63356-9.00004-2

[tjp12961-bib-0081] Lovely RG , Gregor RJ , Roy RR & Edgerton VR (1986). Effects of training on the recovery of full‐weight‐bearing stepping in the adult spinal cat. Exp Neurol 92, 421–435. 3956672 10.1016/0014-4886(86)90094-4

[tjp12961-bib-0082] Magladery JW , Porter WE , Park AM & Teasdall RD (1951). Electrophysiological studies of nerve and reflex activity in normal man. IV. The two‐neuron reflex and identification of certain action potentials from spinal roots and cord. Bull Johns Hopkins Hosp 88, 499–519. 14839348

[tjp12961-bib-0083] Manella KJ , Roach KE & Field‐Fote EC (2013). Operant conditioning to increase ankle control or decrease reflex excitability improves reflex modulation and walking function in chronic spinal cord injury. J Neurophysiol 109, 2666–2679. 23468393 10.1152/jn.01039.2011

[tjp12961-bib-0084] Marsh BC , Astill SL , Utley A & Ichiyama RM (2011). Movement rehabilitation after spinal cord injuries: Emerging concepts and future directions. Brain Res Bull 84, 327–336. 20673791 10.1016/j.brainresbull.2010.07.011

[tjp12961-bib-0085] Martin JH , Choy M , Pullman S & Meng Z (2004). Corticospinal system development depends on motor experience. J Neurosci 24, 2122–2132. 14999063 10.1523/JNEUROSCI.4616-03.2004 PMC6730424

[tjp12961-bib-0086] Martin TA , Keating JG , Goodkin HP , Bastian AJ & Thach WT (1996). Throwing while looking through prisms. I. Focal olivocerebellar lesions impair adaptation. Brain 119, 1183–1198. 8813282 10.1093/brain/119.4.1183

[tjp12961-bib-0087] Matthews PBC (1972). The reflex actions of the muscle receptors In Mammalian Muscle Receptors and their Central Actions, eds Davson H, Greenfield ADM, Whittam R & Brindley GS, pp. 319–409. Williams and Wilkins, Baltimore.

[tjp12961-bib-0088] Mauk MD , Li W , Khilkevich A & Halverson H (2014). Cerebellar mechanisms of learning and plasticity revealed by delay eyelid conditioning. Intern Rev Neurobiol 117, 21–37. 10.1016/B978-0-12-420247-4.00002-6 25172627

[tjp12961-bib-0089] Mendell LM (1984). Modifiability of spinal synapses. Physiol Rev 64, 260–324. 6320234 10.1152/physrev.1984.64.1.260

[tjp12961-bib-0090] Meyer‐Lohmann J , Christakos CN & Wolf H (1986). Dominance of the short‐latency component in perturbation induced electromyographic responses of long‐trained monkeys. Exp Brain Res 64, 393–399. 2948829 10.1007/BF00340475

[tjp12961-bib-0091] Myklebust BM , Gottlieb GL & Agarwal GC (1986). Stretch reflexes of the normal human infant. Dev Med Child Neurol 28, 440–449. 2944785 10.1111/j.1469-8749.1986.tb14281.x

[tjp12961-bib-0092] Myklebust BM , Gottlieb GL , Penn RL & Agarwal GC (1982). Reciprocal excitation of antagonistic muscles as a differentiating feature in spasticity. Ann Neurol 12, 367–374. 7149662 10.1002/ana.410120409

[tjp12961-bib-0093] Neuburger M (1981). Experiments on the reflex mechanism In The Historical Development of Experimental Brain and Spinal Cord Physiology before Flourens, eds Neuberger M & Clarke E, pp. 237–246. The Johns Hopkins University Press, Baltimore.

[tjp12961-bib-0094] Nielsen J , Crone C & Hultborn H (1993). H‐reflexes are smaller in dancers from the Royal Danish Ballet than in well‐trained athletes. Eur J Appl Physiol 66, 116–121. 10.1007/BF01427051 8472692

[tjp12961-bib-0095] Patterson MM (1976). Mechanisms of classical conditioning and fixation in spinal mammals. Adv Psychobiol 3, 381–436. 788481

[tjp12961-bib-0096] Pierrot‐Deseilligny E & Burke D (2012). The Circuitry of the Human Spinal Cord: Spinal and Corticospinal Mechanisms of Movement. Cambridge University Press, Cambridge.

[tjp12961-bib-0097] Perez MA , Lundbye‐Jensen J & Nielsen JB (2007). Task‐specific depression of the soleus H‐reflex after cocontraction training of antagonistic ankle muscles. J Neurophysiol 98, 3677–3687. 17942616 10.1152/jn.00988.2007

[tjp12961-bib-0098] Reinkensmeyer DJ , Guigon E & Maier MA (2012). A computational model of use‐dependent motor recovery following a stroke: optimizing corticospinal activations via reinforcement learning can explain residual capacity and other strength recovery dynamics. Neural Networks 29–30, 60–69. 10.1016/j.neunet.2012.02.002 PMC3678524 22391058

[tjp12961-bib-0099] Riddoch G (1917). The reflex functions of the completely divided spinal cord in man compared with those associated with less severe lesions. Brain 40, 264–402.

[tjp12961-bib-0100] Rossignol S & Frigon A (2011). Recovery of locomotion after spinal cord injury: Some facts and mechanisms. Annu Rev Neurosci 34, 413–440. 21469957 10.1146/annurev-neuro-061010-113746

[tjp12961-bib-0101] Rossignol S , Frigon A , Barrière G , Martinez M , Barthélemy D , Bouyer L , Bélanger M , Provencher J , Chau C , Brustein E , Barbeau H , Giroux N , Marcoux J , Langlet C & Alluin O (2011). Spinal plasticity in the recovery of locomotion. Prog Brain Res 188, 229–241. 21333814 10.1016/B978-0-444-53825-3.00021-8

[tjp12961-bib-0102] Ruigrok TJH , Sillitoe RV , Voogd J (2015). Cerebellum and cerebellar connections In The Rat Nervous System, ed Paxinos G, 4th edn., pp. 133–205. Elsevier, San Diego.

[tjp12961-bib-0103] Sacco K , Cauda F , D'Agata F , Mate D , Duca S & Geminiani G (2009). Reorganization and enhanced functional connectivity of motor areas in repetitive ankle movements after training in locomotor attention. Brain Res 1297, 124–134. 19703428 10.1016/j.brainres.2009.08.049

[tjp12961-bib-0104] Sadtler PT , Quick KM , Golub MD , Chase SM , Ryu SI , Tyler‐Kabara EC , Yu BM & Batista AP (2014). Neural constraints on learning. Nature 512, 423–426. 25164754 10.1038/nature13665 PMC4393644

[tjp12961-bib-0105] Sainburg RL (2015). Should the equilibrium point hypothesis (EPH) be considered a scientific theory? Motor Control 19, 142–148. 25386681 10.1123/mc.2014-0056 PMC5378163

[tjp12961-bib-0106] Sawers A , Allen JL & Ting LH (2015). Long‐term training modifies the modular structure and organization of walking balance control. J Neurophysiol 114, 3359–3373. 26467521 10.1152/jn.00758.2015 PMC4868379

[tjp12961-bib-0107] Schneider C & Capaday C (2003). Progressive adaptation of the soleus H‐reflex with daily training at walking backward. J Neurophysiol 89, 648–656. 12574442 10.1152/jn.00403.2002

[tjp12961-bib-0108] Scholz JP & Schöner G (1999). The uncontrolled manifold concept: identifying control variables for a functional task. Exp Brain Res 126, 289–306. 10382616 10.1007/s002210050738

[tjp12961-bib-0109] Schonewille M , Gao Z , Boele HJ , Vinueza Veloz MF , Amerika WE , Simek AAM , De Jeu MT , Steinberg JP , Takamiya K , Hoebeek FE , Linden DJ , Huganir RL & De Zeeuw CI (2011). Reevaluating the role of LTD in cerebellar motor learning. Neuron 70, 43–50. 21482355 10.1016/j.neuron.2011.02.044 PMC3104468

[tjp12961-bib-0110] Schouenborg J (2008). Action‐based sensory encoding in spinal sensorimotor circuits. Brain Res Rev 57, 111–117. 17920132 10.1016/j.brainresrev.2007.08.007

[tjp12961-bib-0111] Shadmehr R , Smith MA & Krakauer JW (2010). Error correction, sensory prediction, and adaptation in motor control. Ann Rev Neurosci 33, 89–108. 20367317 10.1146/annurev-neuro-060909-153135

[tjp12961-bib-0112] Shmuelof L & Krakauer JW (2011). Are we ready for a natural history of motor learning? Neuron 72, 469–476. 22078506 10.1016/j.neuron.2011.10.017 PMC3389513

[tjp12961-bib-0113] Shurrager PS & Dykman RA (1951). Walking spinal carnivores. J Comp Physiol Psychol 44, 52–62. 10.1037/h0059889 14873849

[tjp12961-bib-0114] Sur M , Nagakura I , Chen N & Sugihara H (2013). Mechanisms of plasticity in the developing and adult visual cortex. Prog Brain Res 207, 243–254. 24309257 10.1016/B978-0-444-63327-9.00002-3

[tjp12961-bib-0115] Suzuki L , Coulon P , Sabel‐Goedknegt EH & Ruigrok TJH (2012). Organization of cerebral projections to identified cerebellar zones in the posterior cerebellum of the rat. J Neurosci 32, 10854–10869. 22875920 10.1523/JNEUROSCI.0857-12.2012 PMC6621006

[tjp12961-bib-0116] Thompson AK , Chen XY & Wolpaw JR (2009). Acquisition of a simple skill: task‐dependent adaptation plus long‐term change in the human soleus H‐reflex. J Neurosci 29, 5784–5792. 19420246 10.1523/JNEUROSCI.4326-08.2009 PMC2696311

[tjp12961-bib-0117] Thompson AK , Pomerantz F & Wolpaw JR (2013). Operant conditioning of a spinal reflex can improve locomotion after spinal cord injury in humans. J Neurosci 33, 2365–2375. 23392666 10.1523/JNEUROSCI.3968-12.2013 PMC3579496

[tjp12961-bib-0118] Thompson AK & Wolpaw JR (2014). Operant conditioning of spinal reflexes: from basic science to clinical therapy. Front Integr Neurosci 8, 25. 24672441 10.3389/fnint.2014.00025 PMC3957063

[tjp12961-bib-0119] Thompson AK & Wolpaw JR (2015). Restoring walking after spinal cord injury: operant conditioning of spinal reflexes can help. Neuroscientist 21, 203–215. 24636954 10.1177/1073858414527541 PMC4167198

[tjp12961-bib-0120] Thompson RF (2005). In search of memory traces. Annu Rev Psychol 56, 1–23. 15709927 10.1146/annurev.psych.56.091103.070239

[tjp12961-bib-0121] Vahdat S , Lungu O , Cohen‐Adad J , Marchand‐Pauvert V , Benali H & Doyon J (2015). Simultaneous brain‐cervical cord fMRI reveals intrinsic spinal cord plasticity during motor sequence learning. PLoS Biol 13, e1002186. 26125597 10.1371/journal.pbio.1002186 PMC4488354

[tjp12961-bib-0122] Wang Y , Pillai S , Wolpaw JR & Chen XY (2006). Motor learning changes GABAergic terminals on spinal motoneurons in normal rats. Eur J Neurosci 23, 141–150. 16420424 10.1111/j.1460-9568.2005.04547.x

[tjp12961-bib-0123] Welsh JP , Yamaguchi H , Zeng XH , Kojo M , Nakada Y , Takagi A , Sugimori M & Llina RR (2005).Normal motor learning during pharmacological prevention of Purkinje cell long‐term depression. Proc Natl Acad Sci U S A 102, 17166–17171. 16278298 10.1073/pnas.0508191102 PMC1288000

[tjp12961-bib-0124] Wolpaw JR (1987). Operant conditioning of primate spinal reflexes: The H reflex. J Neurophysiol 57, 443–458. 3559687 10.1152/jn.1987.57.2.443

[tjp12961-bib-0136] Wolpaw JR (1997). The complex structure of a simple memory. Trends Neurosci 20, 588–594. 9416673 10.1016/s0166-2236(97)01133-8

[tjp12961-bib-0125] Wolpaw JR (2010). What can the spinal cord teach us about learning and memory? Neuroscientist 16, 532–549. 20889964 10.1177/1073858410368314

[tjp12961-bib-0126] Wolpaw JR (2012). Harnessing neuroplasticity for clinical applications. Brain 135, e215. 22374936 10.1093/brain/aws017 PMC3326250

[tjp12961-bib-0127] Wolpaw JR , Braitman DJ & Seegal RF (1983). Adaptive plasticity in the primate spinal stretch reflex: Initial development. J Neurophysiol 50, 1296–1311. 6663327 10.1152/jn.1983.50.6.1296

[tjp12961-bib-0128] Wolpaw JR & Chen XY (2006). The cerebellum in maintenance of a motor skill: a hierarchy of brain and spinal cord plasticity underlies H‐reflex conditioning. Learn Mem 13, 208–215. 16585796 10.1101/lm.92706 PMC1409832

[tjp12961-bib-0129] Wolpaw JR , Herchenroder PA & Carp JS (1993). Operant conditioning of the primate H‐reflex: factors affecting the magnitude of change. Exper Brain Res 97, 31–39. 8131830 10.1007/BF00228815

[tjp12961-bib-0130] Wolpaw JR & Lee CL (1987). Motoneuron response to dorsal root stimulation in anesthetized monkeys after spinal cord transection. Exper Brain Res 68, 428–433. 3480233 10.1007/BF00248809

[tjp12961-bib-0131] Wolpaw JR & Lee CL (1989). Memory traces in primate spinal cord produced by operant conditioning of H‐reflex. J Neurophysiol 61, 563–572. 2709100 10.1152/jn.1989.61.3.563

[tjp12961-bib-0132] Wolpaw JR , O'Keefe JA , Noonan PA & Sanders MG (1986). Adaptive plasticity in the primate spinal stretch reflex: Persistence. J Neurophysiol 55, 272–279. 3950691 10.1152/jn.1986.55.2.272

[tjp12961-bib-0133] Wolpaw JR & Tennissen AM (2001). Activity‐dependent spinal cord plasticity in health and disease. Ann Rev Neurosci 24, 7–43. 10.1146/annurev.neuro.24.1.807 11520919

[tjp12961-bib-0134] Yoon C & Tuszynski MH (2012). Frontiers of spinal cord and spine repair: experimental approaches for repair of spinal cord injury. Adv Exp Med Bio 760, 1–15. 23281510 10.1007/978-1-4614-4090-1_1

[tjp12961-bib-0135] Zehr EP , Hundza SR & Vasudevan EV (2009). The quadrupedal nature of human bipedal locomotion. Exerc Sport Sci Rev 37, 102–108. 19305202 10.1097/JES.0b013e31819c2ed6

